# HIPP1 stabilizes the interaction between CP190 and Su(Hw) in the *Drosophila* insulator complex

**DOI:** 10.1038/s41598-019-55617-6

**Published:** 2019-12-13

**Authors:** Larisa Melnikova, Varvara Molodina, Maksim Erokhin, Pavel Georgiev, Anton Golovnin

**Affiliations:** 1 Department of Drosophila Molecular Genetics, Institute of Gene Biology, Russian Academy of Sciences, 34/5 Vavilov St., 119334 Moscow, Russia; 2 Department of the Control of Genetic Processes, Institute of Gene Biology, Russian Academy of Sciences, 34/5 Vavilov St., 119334 Moscow, Russia

**Keywords:** Molecular biology, Gene regulation

## Abstract

Suppressor of Hairy-wing [Su(Hw)] is one of the best characterized architectural proteins in *Drosophila* and recruits the CP190 and Mod(mdg4)-67.2 proteins to chromatin, where they form a well-known insulator complex. Recently, HP1 and insulator partner protein 1 (HIPP1), a homolog of the human co-repressor Chromodomain Y-Like (CDYL), was identified as a new partner for Su(Hw). Here, we performed a detailed analysis of the domains involved in the HIPP1 interactions with Su(Hw)-dependent complexes. HIPP1 was found to directly interact with the Su(Hw) C-terminal region (aa 720–892) and with CP190, but not with Mod(mdg4)-67.2. We have generated *Hipp1* null mutants *(Hipp*^*Δ1*^) and found that the loss of *Hipp1* does not affect the enhancer-blocking or repression activities of the Su(Hw)-dependent complex. However, the simultaneous inactivation of both HIPP1 and Mod(mdg4)-67.2 proteins resulted in reduced CP190 binding with Su(Hw) sites and significantly altered *gypsy* insulator activity. Taken together, these results suggested that the HIPP1 protein stabilized the interaction between CP190 and the Su(Hw)-dependent complex.

## Introduction

Insulators in *Drosophila* and vertebrate genomes have been identified based on their abilities to disrupt communications between enhancers and promoters, when inserted between them, and to prevent the repression mediated by heterochromatin^1–5^. In the past few years, a special class of architectural proteins has been identified, including some well-known insulator proteins, that are responsible for global chromosomal architecture and the local regulation of enhancer-promoter interactions^6,7^. Insulator proteins are involved in the formation of topologically associated domains, which are the fundamental structural elements of the eukaryotic genome^8–11^.

Today, Suppressor of Hairy-wing [Su(Hw)] is one of the best-characterized insulator proteins in *Drosophila*. The Su(Hw) insulator was first identified in the *gypsy* retrotransposon, which contains a 460-bp sequence with 12 degenerate octamer binding sites for the Su(Hw)^12,13^. The best-characterized insulator complex consists of the Su(Hw) protein and its partners, Mod(mdg4)-67.2 and CP190^14–18^. The CP190 and Mod(mdg4)-67.2 proteins are recruited to chromatin through interactions with each other and with the Su(Hw) protein^19,20^.

The Su(Hw) protein is ubiquitously expressed throughout development and contains an array of 12 C_2_H_2_-type zinc finger (ZF) domains^21,22^. The 6–9 ZF domains specifically recognize a 12-bp motif^23^. In previous genome-wide studies, three classes of Su(Hw) binding regions have been identified, which are characterized by whether they bind Su(Hw) alone (SBS-O), bind both Su(Hw) and CP190 (SBS-C), or bind all three proteins (SBS-CM)^24–27^. CP190 and Mod(mdg4)-67.2 both assist the Su(Hw) complex when binding to SBS-CM sites^19,20^.

The Su(Hw) C-terminal domain (aa 716–892) is responsible for both insulator function^15,21,22^ and the repression of neuronal genes in oocytes^28^. Like the *gypsy* insulator, artificial Su(Hw) protein binding sites can block various enhancers during all stages of *Drosophila* development^29–32^. As a transcriptional repressor, Su(Hw) is necessary for female germline development^33^ and sustained male fertility^34^. Predominantly, only Su(Hw) binds to repressed promoters (SBS-O); therefore, the inactivation of either CP190 or Mod(mdg4)-67.2 does not affect fertility or the Su(Hw)-dependent repression of gene expression in the ovaries^28,35^.

The Mod(mdg4)-67.2 protein, one isoform encoded by the *mod(mdg4)* locus^16,36^, contains an N-terminal BTB domain that forms multimeric complexes^37^. Mod(mdg4)-67.2 interacts with the enhancer-blocking domain of Su(Hw) (716–892 aa), through an isoform-specific C-terminal acidic domain^14,17^, and with the N-terminal region of Su(Hw) (aa 1–238), through the Q-rich domain^19^. Mod(mdg4)-67.2 participates in the enhancer blocking activity of the Su(Hw) complex, although the mechanism of its involvement during insulation remains elusive^14,15,38^.

The CP190 protein contains a BTB/POZ domain at the N terminus, which forms stable homodimers^37,39–41^. CP190 was shown to interact with the N-terminal region of the Su(Hw) protein, located between aa 88 and 202, through its BTB domain^20^. Interestingly, some other known architectural/insulator proteins, such as dCTCF and Pita, also interact with CP190 via its BTB domain^42,43^. CP190 is involved in the recruitment of several complexes to SBS-C and SBS-CM sites, including the nucleosome remodeling factor (NURF), the dimerization partner, RB-like, E2F, and multi-vulval class B (dREAM), and the Spt-Ada-Gcn5 acetyltransferase (SAGA) complexes, which are activators of gene transcription^44–47^.

Recently, HP1 and insulator partner protein 1 (HIPP1) was identified as a potential new partner for Su(Hw)^48^. HIPP1 protein contains a crotonase-fold domain that has been implicated in the transfer of acetyl groups in human chromodomain Y-related (CDY)-like (CDYL) proteins, which are homologous to HIPP1^49,50^, and protein multimerization^51^. Similar to histone acetylation, histone lysine crotonylation is conserved from yeast to human and has been found to be primarily associated with active transcription^52^. Histone crotonylation occurs broadly in all core histones and marks active promoters and enhancers^52^. The CDYL protein negatively regulates histone crotonylation, linking this modification with transcription repression activity^53–56^. HIPP1 is widely expressed during development but is not required for viability or fertility in either sex^57^. The inactivation of the HIPP1 protein does not affect the transcription of Su(Hw)-regulated genes or the activity of the *gypsy* insulator^57^.

To understand the role played by HIPP1 during Su(Hw) insulator functions, we mapped the HIPP1 domains that interact with components of the Su(Hw) complex. We found that HIPP1 binds to the C-terminus of Su(Hw) (aa 637–892), which provides the enhancer-blocking activity of the insulator complex and tissue-specific repression in the ovaries. HIPP1 also interacts with СР190, but not with the Mod(mdg4)-67.2 protein. As previously demonstrated^57^, the inactivation of *Hipp1* does not affect fly fertility or Su(Hw) activity. However, the simultaneous inactivation of *Hipp1* and *mod(mdg4)-67*.*2* led to significantly altered *gypsy* insulator function and strongly reduced the recruitment of CP190 to SBS-CM sites. Therefore, the results of this study suggested that the recruitment of HIPP1 and CP190 to the Su(Hw) complexes are interdependent.

## Results

### HIPP1 directly interacts with the Su(Hw) and CP190 components of the insulator complex

**Figure 1:**
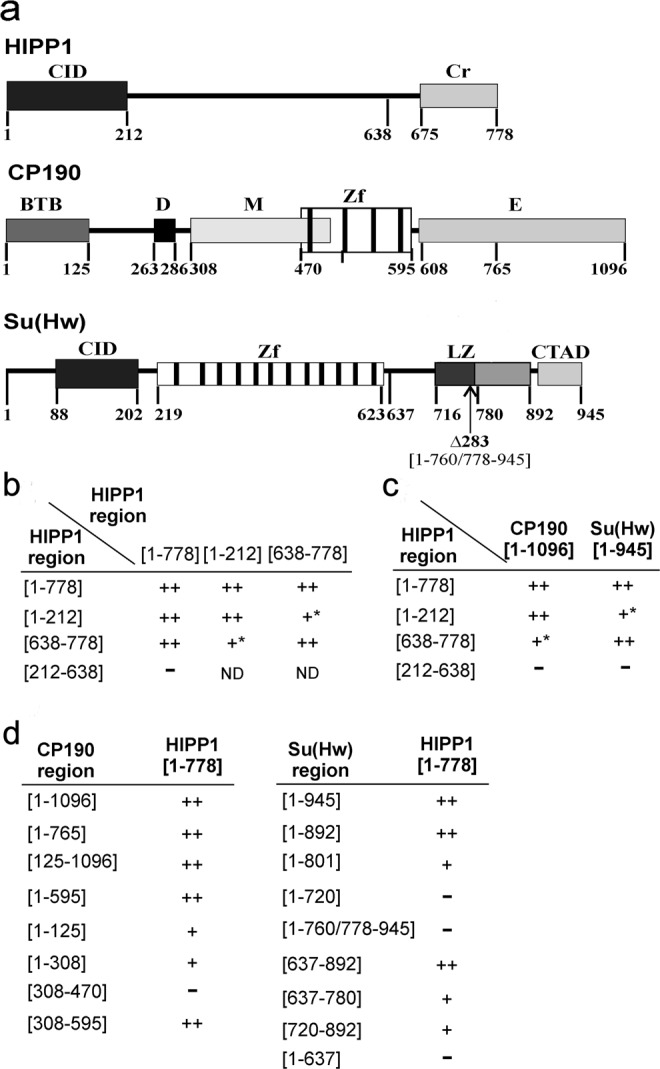
Summary of the interactions between HIPP1 and insulator proteins in the yeast two-hybrid assay. (**a**) Structural scheme of HIPP1, CP190, and Su(Hw) proteins, showing their domains and the corresponding numbers of the amino acid residues: CID, CP190 interaction domain; Cr, crotonase-fold domain; BTB, BTB/POZ domain; D, aspartic acid-rich (D-rich) domain; M, centrosomal targeting domain; E, C-terminal glutamic acid-rich domain; Zf, zinc-finger domain; LZ, leucine zipper motif; CTAD, C-terminal acidic domain. The vertical arrow under the scheme of the Su(Hw) protein indicates the Su(Hw)^Δ283^ mutation. (**b**) The results of testing interactions between HIPP1 domains. (**c**) The results of testing HIPP1 domains for interactions with CP190 and Su(Hw) proteins. (**d**) The results of testing CP190 and Su(Hw) domains for interactions with HIPP1 protein. All experiments were performed in triplicate. Numbers in brackets indicate amino acid residues flanking the protein regions included in analysis. The plus signs indicate the relative strength of two-hybrid interactions (Fig. S1); the minus sign indicates the absence of the interaction; the asterisk indicates that the interaction was observed only when the DBD of GAL4 was fused to the C-terminal region of HIPP1 derivatives.

Because interactions between HIPP1 and insulator proteins have not yet been sufficiently studied, we tested these interactions using the yeast two-hybrid assay (Y2H) (Figs. 1 and S1). HIPP1 was found to interact with both Su(Hw) and CP190 proteins, but not with Mod(mdg4)-67.2.

Our next aim was to identify the HIPP1 domains involved in protein-protein interactions. According to secondary structure prediction^58^, HIPP1 contains two highly structured regions at the N and C termini (aa 1–212 and 675–778, respectively) (Figs. 1a and S2), with the C-terminal region corresponding to the crotonase domain^49,50^. Based on these predictions, we dissected HIPP1 into domains and then studied the protein interactions with these domains using Y2H (Fig. 1b). We found that HIPP1 can form dimers through both its crotonase and N-terminal domains. Moreover, weak interaction between these domains was observed. The interactions found with Y2H were further verified by a GST pull-down assay (Fig. S3a,b). Thus, HIPP1 has the potential to form multimers. Additionally, tests for the interactions between HIPP1 domains and the insulator proteins (Fig. 1c) showed that the N-terminal domain interacts with CP190, whereas the crotonase domain interacts with Su(Hw). These interactions were also verified in GST pull-down experiments (Fig. S3b).

The CP190 protein includes a cluster of four ZF C_2_H_2_ domains: the BTB/POZ domain at the N-terminus, a glutamic acid-rich (E-rich) domain at the C-terminus, with aspartic acid-rich (D-rich) and centrosomal (M) domains between them^39,59,60^ (Fig. 1a). We found that the M domain (aa 308–470), together with the ZF domain (aa 470–595), is required for the interaction of CP190 with HIPP1 (Fig. 1d). Moreover, the BTB domain of CP190 interacts weakly with HIPP1.

Next, we found that the Su(Hw) region from aa 637 to 892 is required for its interaction with HIPP1 (Fig. 1d), and this interaction was reduced when smaller Su(Hw) regions were tested (i.e., aa 637–780 or 720–892). The deletion of aa 760–778 (resembling the Su(Hw)^Δ283^ mutation) completely abolished the interaction with HIPP1. The results obtained with Y2H were confirmed by a GST pull-down assay (Fig. S3b). Thus, HIPP1 interacts with the Su(Hw) region that is involved in enhancer-blocking and repression activity.

Finally, we tested the interaction between the HIPP1 and Mod(mdg4)-67.2 proteins in GST pull-down assays (Fig. S3c). In this analysis, as in Y2H, these proteins did not interact with each other. Thus, HIPP1 does not directly interact with Mod(mdg4)-67.2.

To further confirm these results, we performed co-immunoprecipitation (co-IP) assays between HIPP1 and FLAG-tagged components of the Su(Hw) complex, which were expressed in S2 cells (Fig. S4a). The results showed that HIPP1 co-immunoprecipitated with all of the tested proteins, including Su(Hw), CP190, and Mod(mdg4)-67.2. The co-IP between HIPP1 and Mod(mdg4)-67.2 appeared to have been mediated by Su(Hw) and CP190 (Fig. S3c). A similar explanation is possible for the observed co-IP between HIPP1 and FLAG-tagged Su(Hw)^Δ283^ (Fig. S4b). A FLAG-tagged CP190 protein lacking the BTB domain co-immunoprecipitated with HIPP1 to a similar extent as the full-length protein, indicating the minor role played by the BTB domain in this interaction. The co-IP between HIPP1 and FLAG-tagged CP190 lacking the M domain (aa 308–470) was significantly reduced compared with the full-length CP190 protein, which confirmed the critical role played by this domain in the CP190–HIPP1 interaction (Fig. S4b).

### Both Su(Hw) and CP190 are required for HIPP1 binding to SBSs

**Figure 2:**
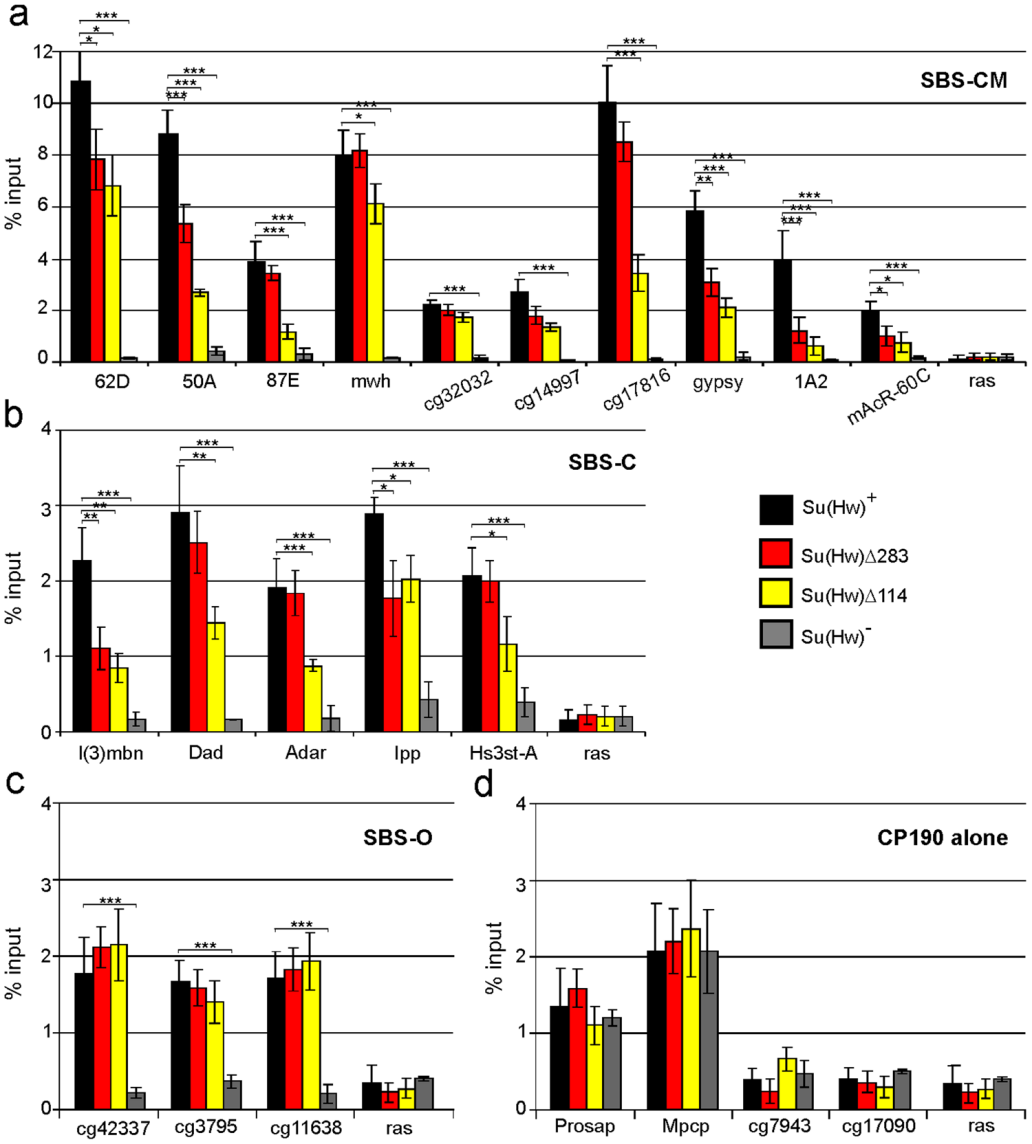
Binding of HIPP1 protein to genomic sites in pupae from transgenic lines expressing full-length Su(Hw)^+^, Su(Hw)^Δ283^, and Su(Hw)^Δ114^. (**a**) SBS-CM sites; (**b**) SBS-C sites; (**c**) SBS-O sites; (**d**) CP190 alone sites. Su(Hw)^−^ refers to the *y*^*2*^ *w*^*1118*^; *su(Hw)*^*v*^/*su(Hw)*^*e04061*^ background. The different variants of the Su(Hw) protein were expressed in the *y*^*2*^*sc*^*D1*^*ct*^*6*^; *P{Su(Hw)}-38D*/*P{Su(Hw)}-38D*; *su(Hw)*^*v*^/*su(Hw)*^*e04061*^ lines, where P{Su(Hw)} are Su(Hw)^+^ − *P{w*^+^*; UbqW-Su(Hw)1-945-FLAG}*; Su(Hw)Δ114 − *P{w*^+^*; UbqW-Su(Hw)1-88*/*202-945-FLAG};* Su(Hw)Δ283 − *P{w*^+^*; UbqW-Su(Hw)1-760*/*778-945-FLAG}*. The *ras64B* coding region (ras) was used as a control devoid of Su(Hw) binding sites. The percent recovery of immunoprecipitated DNA (Y-axis) was calculated relative to the amount of input DNA. Error bars indicate standard deviation of three independent biological replicates. Asterisks indicate significance levels (Student’s *t*-test) of *p < 0.05, **p < 0.01, or ***p < 0.001. P values are not displayed for significance >0.05. Significance levels are shown for treatment comparisons indicated by horizontal brackets (here and in Figs. 4–6). ChIP was performed with antibodies against HIPP1. Statistical analysis was performed relative to the Su(Hw)+ variant.

To reveal the roles played by the CP190 and Su(Hw) proteins during HIPP1 recruitment to SBSs, we compared the binding of HIPP1 in pupae from transgenic lines expressing either Su(Hw)^+^, Su(Hw)^Δ283^ or Su(Hw)^Δ114^ in the *su(Hw)*^*–*^ [*su(Hw)*^*v*^/*su(Hw)*^*e04061*^ *trans*-heterozygous] background (Figs. 2a–d and S5a–d). As a model system, we used ten SBS-CM sites, including the known insulator sites 62D, 50A, 87E, 1A2 and *gypsy*, in addition to a site from the *mAcR-60C* promoter, which Su(Hw) represses in the ovaries (Figs. 2a and S5a). In addition, we tested five SBS-C sites, three SBS-O sites, and four sites through which CP190 binds to chromatin independent of Su(Hw) (Figs. 2b–d and S5b–d). Chromatin immunoprecipitation (ChIP)-quantitative polymerase chain reaction (qPCR) showed that in the *su(Hw)*^*–*^ background, HIPP1 was not recruited to SBSs (Fig. 2a–c) confirming that HIPP1 cannot bind to SBSs in the absence of the Su(Hw) protein^57^.

The mutant Su(Hw)^Δ283^ protein has a 16-aa deletion (from aa 760 to 778), which is critical for the enhancer-blocking and repressive activities of Su(Hw)^21,22,28^. The Su(Hw)^Δ283^ mutant was unable to interact with HIPP1 in the Y2H assay; however, in the Su(Hw)^Δ283^ line, HIPP1 was still able to bind to all tested SBSs (Fig. 2a–c). The results of Y2H and co-IP assays suggested that HIPP1 may be recruited to chromatin through the interaction with CP190. The BTB domain of CP190 binds to the two regions located between aa 88 and 202 at the N-terminus of Su(Hw). The deletion of these regions in the Su(Hw)^Δ114^ mutants prevented CP190 recruitment to the Su(Hw) complex^20^. The decreased CP190 occupancy on the SBC-CM and SBS-C sites in Su(Hw)^Δ114^ (Fig. S5a,b) resulted in the reduction of HIPP1 enrichment (Fig. 2a,b). In summary, these data further confirm that the HIPP1 protein is recruited to SBSs through direct interactions with Su(Hw) and CP190 insulator components.

It is important, not even the Su(Hw)^Δ283^ mutation appeared to affect HIPP1 binding to the SBS-О (Fig. 2с), where Su(Hw) binds HIPP1 independent of CP190 (Fig. S5с). An unknown protein (instead of CP190) may bind to the SBS-O sites in complex with Su(Hw) to facilitate the recruitment of HIPP1 to these genome regions. In addition, HIPP1 was recruited to the two of the four tested CP190 standalone sites: Prosap and Mpcp (Fig. 2d). As expected, at these sites, HIPP1 binding was unaffected by the mutant Su(Hw)^Δ114^ and Su(Hw)^Δ283^ proteins or in the *su(Hw)*^*–*^ background (Fig. 2d). Thus, CP190 can recruit HIPP1 to chromatin independent of Su(Hw).

### Simultaneous inactivation of *Hipp1* and *mod(mdg4)-67*.*2* affects the function of the *gypsy* insulator

To directly test the role played by HIPP1 in the Su(Hw)-dependent complex, we used the genome editing CRISPR/Cas9 approach to create a null mutation in the gene encoding HIPP1 (Fig. S6a). A 286-bp sequence, including the transcription initiation site and the ATG start codon, was deleted from the *Hipp1* gene and substituted with a *lox*-flanked *dsRed* reporter, under the control of the 3xPE promoter^61,62^. After removing the *dsRed* reporter through Cre-mediated recombination, the fragment containing the deletion was cloned by PCR and sequenced (Fig. S6b). Flies homozygous for the mutant *Hipp*^*Δ1*^ gene were viable, fertile, and had wild-type (wt) phenotypes. The absence of the HIPP1 protein in these flies was confirmed by Western blot analysis (Fig. S6c).

**Figure 3:**
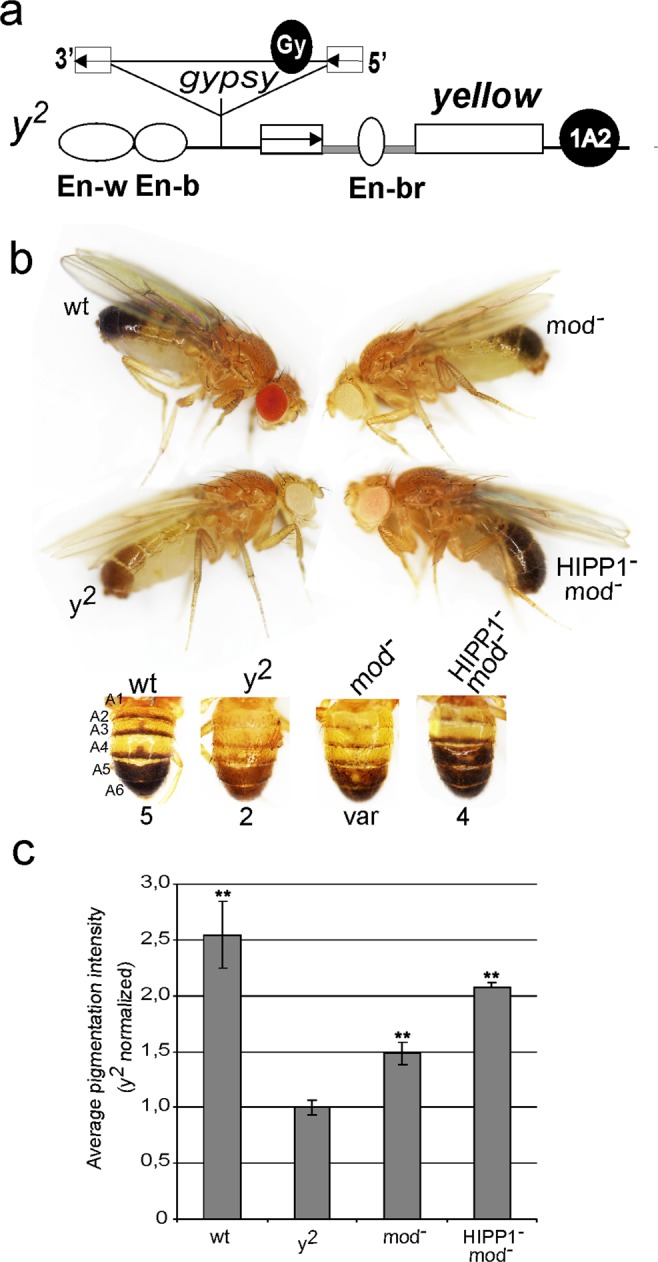
Effects of the combination of *Hipp*^*Δ1*^and *mod(mdg4)*^*u1*^ mutations on *gypsy* enhancer blocking activity. (**a**) Scheme (not to scale) of the *y*^*2*^ allele. Exons in the *yellow* gene are shown as white rectangles; the *yellow* wing (En-w) and body (En-b) enhancers are shown as partially overlapping gray boxes; the bristle enhancer (En-br) is shown as a gray oval in the *yellow* intron. The transcription start site is indicated by an arrowhead. The *gypsy* insertion is shown as a triangle, in which the black circle marked Gy is the *gypsy* insulator and the white boxes are long terminal repeats, with arrows indicating their directions. (**b**) Images show the effects of the *mod(mdg4)*^*u1*^/*mod(mdg4)*^*u1*^ − [mod^*−*^], *Hipp*^*Δ1*^/*Hipp*^*Δ1*^ − [HIPP1^*−*^], and *Hipp*^*Δ1*^*mod*(*mdg4*)^*u1*^/*Hipp*^*Δ1*^*mod*(*mdg4*)^*u1*^ − [HIPP1^*−*^ mod^*−*^] mutants on the *yellow* phenotype and on abdomen pigmentation in three-day-old *y*^*2*^ males. The term “wt” refers to the wild type. Numbers show the *yellow* expression scores in the abdominal segments (5, maximal pigmentation; 2, weak pigmentation; var, variegated pigmentation; 4, moderate pigmentation). (**c**) Quantitative assessment of the pigmentation intensity of the abdominal A5 segments. Images were analysed using the Measure tool in Fiji. “Mean” parameters were used for histogram generation. For each genotype, 20 representative images were processed. Pigmentation intensity (Y axis) is shown as fold change relative to the *y*^*2*^ variant. Error bars indicate standard deviation of pigmentation intensity. Asterisks indicate significance levels (Student’s *t*-test) of **p < 0.01. Statistical analysis was performed relative to the *y*^*2*^ variant.

Deletions previously performed in the *Hipp1* gene did not reduce the enhancer-blocking function of the *gypsy* insulator in the *y*^2^ allele^57^. In the *y*^2^ mutation (Fig. 3a), *gypsy* is inserted between the promoter of the *yellow* gene and the distal enhancers responsible for *yellow* expression in the wings and body cuticle^63^. In this case, the Su(Hw)-dependent insulator complex blocks only the distal enhancers (flies display a yellow colour in the body and wings) but not the bristle enhancer (black bristles, *y*^2^ phenotype, Fig. 3b), which is placed in the intron of the gene^63,64^. The newly generated *Hipp*^*Δ1*^ mutation also did not affect the *gypsy* insulator activity in the *y*^2^ allele.

The *mod(mdg4)*^*u1*^ allele generates a mutant Mod(mdg4)-67.2 protein, which does not interact with the Su(Hw) insulator^17^. The *mod(mdg4)*^*u1*^ mutation affects the *y*^2^ phenotype by converting the *gypsy* insulator into a repressor of *yellow* transcription in the bristles and in some cuticle cells^15,16^. At the same time, the enhancer-blocking activity of the insulator is partially lost. Therefore, the abdominal segments of *y*^2^ males show a variegated cuticular phenotype in the *mod*(*mdg4*)^*u1*^ background (Fig. 3b). In some cuticle cells, insulator activity was significantly reduced, resulting in restoration of *yellow* expression; in other cells, the effects of the *gypsy* insulator were enhanced due to the direct repression of *yellow* transcription.

Previously, we demonstrated that multiple interactions between Mod(mdg4)-67.2, CP190 and Su(Hw) were involved in the formation of the insulator complex on SBS-CM sites^20^. Redundancy among protein interactions may mask the role played by HIPP1 during the organization of the insulator complex. Therefore, we examined the effects of *Hipp*^*Δ1*^ on the activity of the *gypsy* insulator in the *mod*(*mdg4*)^*u1*^ mutant background. To minimize background effects, three independent *y*^2^; *Hipp*^*Δ1*^*mod*(*mdg4*)^*u1*^/*Hipp*^*Δ1*^*mod*(*mdg4*)^*u1*^ lines were generated. In all of the obtained lines, the combination of *Hipp*^*Δ1*^ and *mod(mdg4)*^*u1*^ mutations considerably restored *yellow* expression in the body and wings (Fig. 3b,c). Thus, the simultaneous loss of Mod(mdg4)-67.2 and HIPP1 proteins considerably affected the enhancer blocking activity of the *gypsy* insulator.

To further confirm that the combination of *Hipp*^*Δ1*^ and *mod(mdg4)*^*u1*^ was responsible for the weakening of *gypsy* insulator activity, we tested whether the *Hipp1*+ or *Mod(mdg4)-67*.*2* + transgenes could compensate for the observed effects in the *y*^2^; *Hipp*^*Δ1*^*mod*(*mdg4*)^*u1*^/*Hipp*^*Δ1*^*mod*(*mdg4*)^*u1*^ line. For this purpose, we used transgenic lines carrying constructs that expressed Mod(mdg4)-67.2 or HIPP1 under the control of the UAS promoter. To induce UAS expression, the tested lines were crossed with the transgenic line *y*^2^; Act5C-GAL4/CyO; *Hipp*^*Δ1*^*mod*(*mdg4*)^*u1*^/*Hipp*^*Δ1*^*mod*(*mdg4*)^*u1*^, which carries the GAL4 gene, under the control of the Act5C promoter, on the second chromosome. Phenotypic analyses of males showed that the expression of Mod(mdg4)-67.2 completely restored the mutant *y*^2^ phenotype, whereas the expression of HIPP1 reverted the mutants to the *y*^2^*; mod(mdg4)*^*u1*^/*mod(mdg4)*^*u1*^ phenotype (Fig. S7a,b). These results confirmed the existence of a functional interaction between *Hipp*^*Δ1*^ and *mod*(*mdg4*)^*u1*^.

### HIPP1 participates in the recruitment of CP190 to SBSs

**Figure 4:**
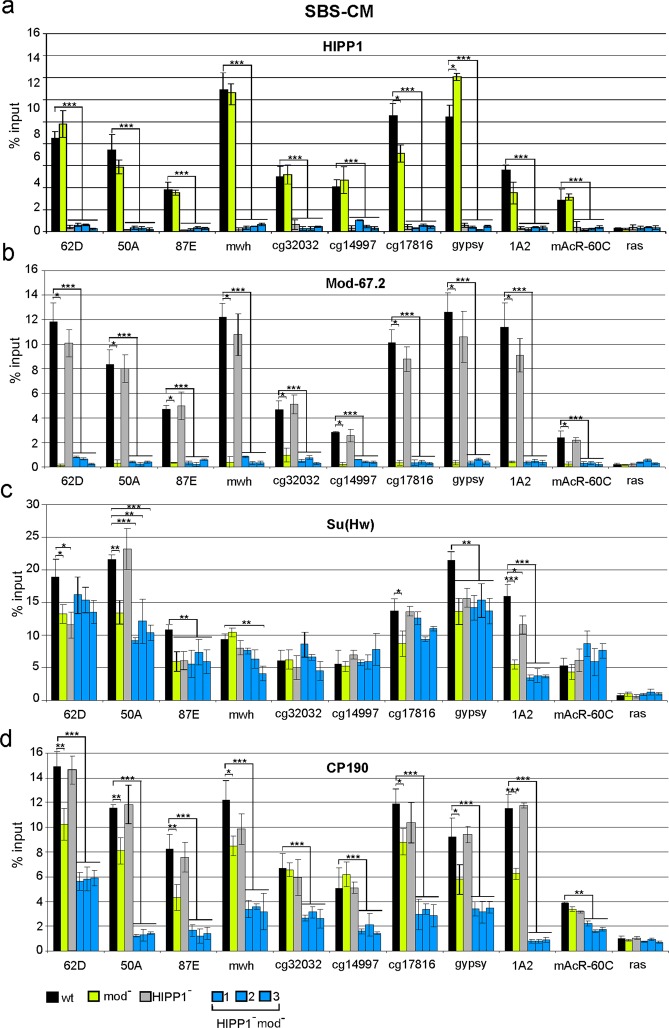
Binding of HIPP1 and insulator proteins to SBS-CMs in pupae from lines bearing *mod(mdg4)*^*u1*^ and *Hipp*^*Δ1*^ mutations. ChIP was performed with antibodies against (**a**) HIPP1; (**b**) Mod(mdg4)-67.2; (**c**) the Su(Hw) N-terminal domain; and (**d**) CP190. The lines used in this analysis are designated as [wt] − *y*^*2*^*w*^*1118*^, [mod^*−*^] − *y*^*2*^*w*^*1118*^, *mod*(*mdg4*)^*u1*^/*mod*(*mdg4*)^*u1*^, [HIPP1^*−*^] − *y*^*2*^*w*^*1118*^;*Hipp*^*Δ1*^/*Hipp*^*Δ1*^, and [HIPP1^−^ mod^−^] − *y*^*2*^*w*^*1118*^;*Hipp*^*Δ1*^*mod*(*mdg4*)^*u1*^/*Hipp*^*Δ1*^*mod*(*mdg4*)^*u1*^. Statistical analysis was performed relative to the wild type condition. Other designations are as described in Fig. 2.

To understand how the simultaneous inactivation of HIPP1 and Mod(mdg4)-67.2 affected the binding of Su(Hw) and CP190 to chromatin, we performed ChIP analyses on pupae from wt, *mod*(*mdg4*)^*u1*^/*mod*(*mdg4*)^*u1*^ (*mod*^*−*^), and *Hipp*^*Δ1*^/*Hipp*^*Δ1*^ (*HIPP1*^*−*^) lines and three *Hipp*^*Δ1*^ *mod*(*mdg4*)^*u1*^/*Hipp*^*Δ1*^ *mod*(*mdg4*)^*u1*^ (*HIPP1*^*−*^*mod*^*−*^) lines. First, we tested ten SBS-CM sites, including the *gypsy* insulator (Fig. 4a–d). HIPP1 was detected at all sites in the wt, but not in the *Hipp*^*Δ1*^ background, which confirmed the specificity of our antibodies (Fig. 4a). ChIP-qPCR in wt and *Hipp*^*Δ1*^ pupae also showed that HIPP1 inactivation did not affect the binding among the Su(Hw), CP190, and Mod(mdg4)-67.2 proteins (Fig. 4a–d). Thus, the HIPP1 protein itself does not play a major role in the recruitment of the other components of the insulator complex to SBS-CMs.

ChIP-qPCR analysis in *mod*^*−*^ pupae showed that the Mod(mdg4)^u1^ protein did not bind to the tested SBSs (Fig. 4b). In the absence of Mod(mdg4)-67.2, the binding of HIPP1 to the selected sites was unchanged; however, the binding of Su(Hw) and CP190 to some sites was reduced. This result confirmed the previously obtained data regarding the role played by Mod(mdg4)-67.2 during the recruitment of the Su(Hw)-dependent complex to chromatin^20^ and showed that Mod(mdg4)-67.2 is not involved in the recruitment of HIPP1 to SBSs.

The simultaneous inactivation of the HIPP1 and Mod(mdg4)-67.2 proteins in the mutant *HIPP1*^*–*^*mod*^*–*^ lines weakened Su(Hw) binding to SBS to the same extent as the *mod*(*mdg4*)^*u1*^ mutation. Surprisingly, the binding of CP190 was dramatically decreased for half of the tested sites, whereas binding was completely abolished for the other half (Fig. 4c). These results suggested that the Mod(mdg4)-67.2 and HIPP1 proteins cooperatively recruit CP190 to SBS-CMs.

**Figure 5:**
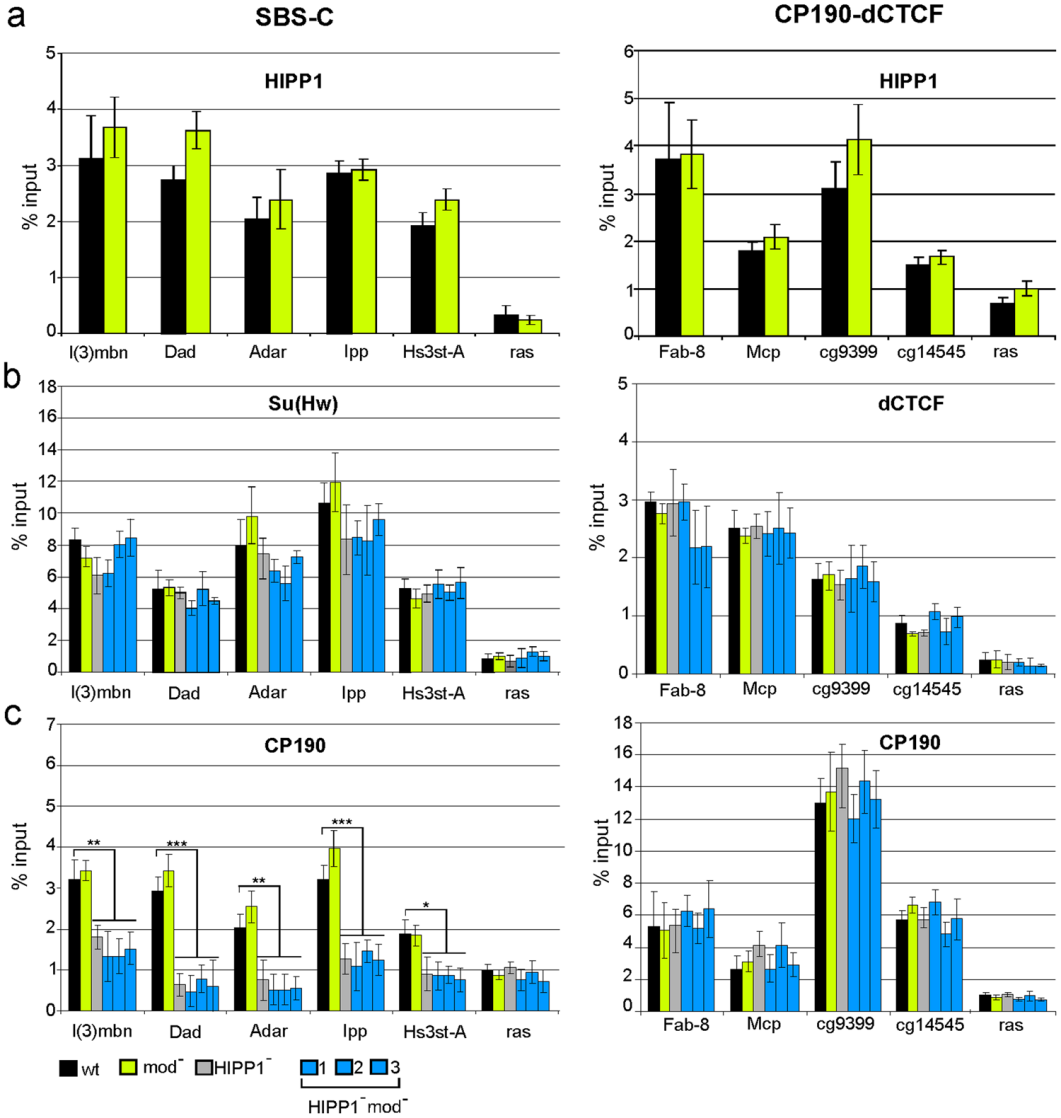
Binding of HIPP1 and insulator proteins to SBS-Cs and CP190-dCTCFs in pupae from lines bearing *mod(mdg4)*^*u1*^ and *Hipp*^*Δ1*^ mutations. (**a**) HIPP1 binding; (**b**) Su(Hw) and dCTCF binding; (**c**) CP190 binding. Designations of the analyzed lines are as described in Fig. 4. ChIP was performed with antibodies against HIPP1, the Su(Hw) N-terminal domain, CP190, and dCTCF. Statistical analysis was performed relative to the wild type condition. Other designations are as described in Fig. 2.

Next, we studied the effects of HIPP1 inactivation on the recruitment of the Su(Hw) and CP190 proteins to other types of genomic sites. We used five SBS-C sites, four sites bound by dCTCF and CP190, five standalone Su(Hw) sites, and four standalone CP190 sites. ChIP-qPCR analyses showed that HIPP1 binds to all SBS-C, CP190-dCTCF, and SBS-O sites and to two of the four CP190 alone sites (Figs. 5a and S8a). In accordance with the absence of Mod(mdg4)-67.2 binding to the selected sites, the *mod*(*mdg4*)^*u1*^ mutation did not affect the binding of the HIPP1, Su(Hw), and CP190 proteins to these sites (Figs. 5a–c and S8a,b). As with SBS-CMs, the Su(Hw) binding to SBS-C and SBS-O sites remained almost unchanged compared with the wt, both in the *mod* ^*–*^ line and in the *HIPP1*^*–*^*mod*^*–*^ line (Figs. 5b and S8b). The binding of the dCTCF protein to CP190-dCTCFs in mutant lines was also not affected (Fig. 5b). However, unlike SBS-CMs, even the inactivation of only HIPP1 protein in the *HIPP1*^*–*^ line resulted in the complete loss of CP190 binding to most of the tested SBS-C sites (Fig. 5c). Interestingly, we did not observe any reductions in CP190 binding to either the control CP190 standalone sites or the CP190-dCTCF sites (Figs. 5c and S8b). Thus, the HIPP1 protein is only essential for the preferential recruitment of CP190 to Su(Hw) binding sites.

### The combination of *Hipp*^Δ1^ and *mod(mdg4)*^*u1*^ mutations did not affect Su(Hw)-dependent repression in ovaries

The Su(Hw) protein is involved in the repression of many neural genes in the ovaries^33,35^. Previously published data showed that neither HIPP1 nor Mod(mdg4)-67.2 proteins play significant roles in tissue-specific Su(Hw)-dependent repression^28,35,57^. However, we found that the combination of the *Hipp*^*Δ1*^ and *mod*(*mdg4*)^*u1*^ mutations affected the insulator activity of the Su(Hw) complex. In addition, both proteins interact with the C-terminal domain (aa 720–892) of Su(Hw), which is responsible for promoter repression^28^. Therefore, we decided to test whether the simultaneous inactivation of HIPP1 and Mod(mdg4)-67.2 affects Su(Hw)-dependent repression in the ovaries.

**Figure 6:**
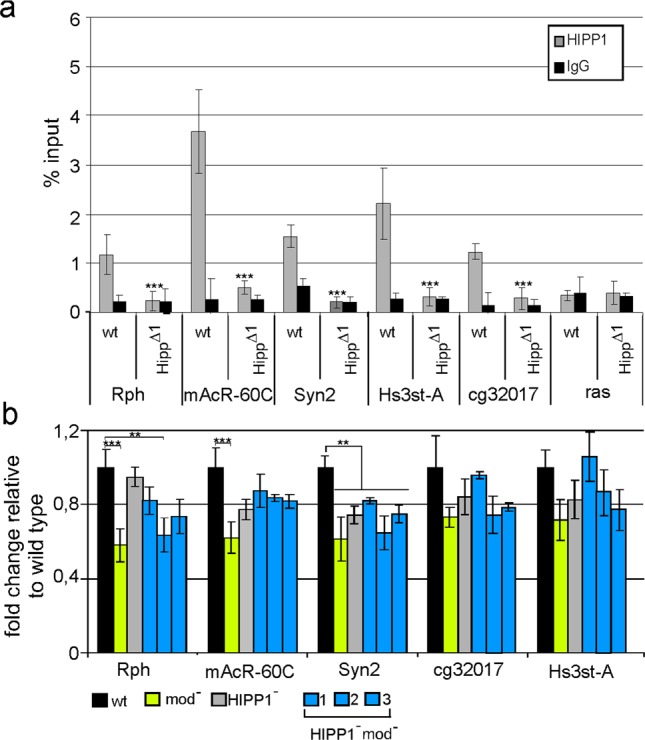
HIPP1 and Mod(mdg4)-67.2 did not affect Su(Hw)-dependent repression in ovaries. (**a**) ChIP-qPCR data showing HIPP1 binding to model promoters in the wild type (wt) and *Hipp*^*Δ1*^ pupae. ChIP was performed with antibodies against HIPP1. IgG refers to nonspecific immunoglobulins. Statistical analysis was performed relative to the wild type condition. Other designations are as described in Fig. 2. (**b**) RT-qPCR data showing the expression of model genes in the ovaries of wt and mutant females. Expression levels are shown as the fold-change relative to wt levels. Error bars indicate standard deviations of two independent biological replicates. The lines used in this analysis are designated as described in Fig. 4.

We used the model system described earlier, comprised of five representative neural genes whose promoters are bound by Su(Hw) alone (*Rph*, *cg32017*, and *Hs3st-A*), by Su(Hw) and CP190 (*Syn2*), or by Su(Hw), CP190, and Mod(mdg4)-67.2 (*mAcR-60C*)^28^. These genes are repressed in the ovaries and are actively transcribed in the heads. The binding of HIPP1 to these model promoters was evaluated using ChIP-qPCR analysis. As expected, HIPP1 was recruited to the selected sites in the wt, but not in the *Hipp1*^*Δ1*^ mutant background (Fig. 6a).

To determine the expression levels of the model genes, we performed real-time (RT)-qPCR analyses of their activities in the ovaries of wt and mutant females (Fig. 6b). The transcription levels of the model genes did not increase in any model line compared with those in the wt. This result confirmed that the HIPP1 and Mod(mdg4)-67.2 proteins are not involved in the Su(Hw)-dependent repression in ovaries.

## Discussion

Previous studies have identified HIPP1 as a new partner of HP1a and Su(Hw) proteins^48,57^. Here, we have mapped the regions of the HIPP1 protein that are responsible for its interaction with the Su(Hw) insulator complex components. The C-terminal crotonase-fold domain of HIPP1 interacts with the Su(Hw) region (aa 637–892) involved in enhancer blocking and repression, whereas the N-terminal domain interacts with the central CP190 region (aa 308–595). We have found that HIPP1 is effectively recruited to SBSs through interactions with both Su(Hw) and CP190. The HIPP1-Su(Hw) interaction is apparently stabilized by CP190, as demonstrated by the strong reduction in HIPP1 association with the insulator complex in Su(Hw)^Δ114^ mutants.

However, in our experiments, as in previous experiments^57^, the inactivation of HIPP1 alone did not affect the *gypsy* enhancer blocking activity. In the absence of Mod(mdg4)-67.2, the *gypsy* insulator acts as a silencer that represses *yellow* expression^15,16^. Previously, we suggested that Mod(mdg4)-67.2 competes with a hypothetical repressive complex that binds to the C-terminal region of Su(Hw)^28^. In the *y*^*2*^ model system, the combination of *mod(mdg4)*^*u1*^ and *Hipp*^*Δ1*^ mutations weakens *gypsy* insulator activity in the body and wings, but does not affect is repressive activity in bristles. HIPP1 was associated with the promoters of all of the neural genes that are negatively regulated by Su(Hw) that were tested in this study. However, *Hipp*^*Δ1*^ alone or in combination with *mod(mdg4)*^*u1*^ did not affect either the recruitment of Su(Hw) to chromatin or the transcription of model genes in the ovaries. Taken together, these results showed that HIPP1 is either not a component of the repressive complex or that its repressive activity is redundant.

**Figure 7:**
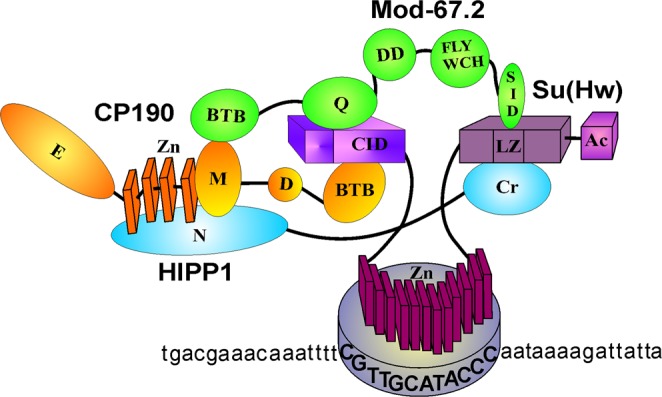
Scheme of the protein-protein interactions involved in the formation of the Su(Hw)-dependent complex. The CP190 domains are shown as yellow ovals, with four zinc fingers shown as yellow parallelepipeds; the Mod(mdg4)-67.2 domains are shown as green ovals; the HIPP1 domains are shown as blue ovals; the Su(Hw) domains are shown as lilac parallelepipeds. Bold capital letters indicate the Su(Hw) binding site. The domain abbreviations are as described in Fig. 1.

Here, we found that HIPP1 and Mod(mdg4)-67.2 cooperatively facilitate the recruitment of CP190 to the insulator complex. Our previous results provided compelling evidence for a high degree of complexity among the interactions between the insulator proteins, which are required to form the Su(Hw) insulator complexes^19,20^. The stability of the complex is ensured by the multiplicity of interactions among Su(Hw), CP190 and Mod(mdg4)-67.2. HIPP1 appears to be a novel component of the Su(Hw) insulator complex that promotes the recruitment of CP190, which, in turn, enhances the binding of HIPP1 with the complex (Fig. 7). The inactivation of a single protein in the complex, either HIPP1 or Mod(mdg4)-67.2, only partially disrupts the CP190-Su(Hw) interaction. However, in the absence of both proteins, CP190 remains bound to Su(Hw) only by its BTB domain, which leads to the reduced association between these proteins.

The role of HIPP1 during the recruitment of CP190 was most pronounced for SBS-Cs, to which Su(Hw) binds only in combination with CP190. In the absence of HIPP1, the binding of CP190 to SBS-Cs was critically reduced, which was not observed at the CP190-dCTCF sites. Thus, in the absence of Mod(mdg4)-67.2, HIPP1 appeared to be critical for the stabilization of the Su(Hw)-CP190 complex. Importantly, HIPP1 was essential for the recruitment of CP190 only to the Su(Hw) sites, but not to the dCTCF sites or to sites bound by unknown proteins. This can only be explained by the existence of a specific interaction between the HIPP1 protein and Su(Hw), but not with other architectural proteins. It has been shown that in *Drosophila* interphase cell nuclei, the Su(Hw), Mod(mdg4)-67.2, and CP190 proteins colocalize in speckles, named insulator bodies^18,65,66^. We suggest that Su(Hw)-dependent complexes are formed in insulator bodies, and then pre-assembled complexes are bound to chromatin^20,66^. Since HIPP1 is extensively colocalized with Su(Hw) in nuclei^57^, it is likely that these proteins are pre-assembled in complexes before binding to chromatin.

A previous study^57^ suggested that HIPP1 contains a functional crotonase domain. This domain, which was demonstrated to have crotonyl CoA hydratase function, is involved in the negative regulation of histone lysine crotonylation^54,56^, a histone modification that is associated with active transcription. Thus, HIPP1 might be involved in the repression of transcription. Interestingly, HIPP1 interacts with the Su(Hw) domain that is involved in the repression of promoters and insulation. However, both here and in the study by Glen and Geyer (2019), the inactivation of HIPP1 clearly did not affect the insulator or repression activities of the Su(Hw) complexes. HIPP1 belongs to a family of at least 17 proteins with predicted crotonase-like fold domains^57^. Thus, the function of HIPP1 could be compensated by another protein with the same activity.

In summary, our results provided evidence for the structural role played by the HIPP1 protein during the formation of Su(Hw) complexes involving CP190. Further studies are necessary to better understand the role played by HIPP1 during transcriptional regulation and insulation.

## Materials and Methods

The constructs used for yeast two-hybrid and GST pull-down assays, expression in S2 cells, and transgenic constructs are described in the Supplementary Materials.

### Germ-line transformation, genetic crosses, and phenotypic analysis

All flies were maintained at 25 °C, on standard yeast medium. To obtain flies with deletion in the *Hipp1* gene, the DNA of reporter constructs was injected into preblastoderm embryos with the *y*^*1*^ *M{w*^+*mC*^ = *nos-Cas9*.P}ZH-2A w* genotype^62^. The resulting flies were crossed with *y*^*2*^*w*^*1118*^ flies, and the progeny carrying the *Hipp*^*Δ1*^ mutation was identified by *dsRed* expression.

To obtain transgenic flies with the insertion of P{w^+^; 5xUAS hsp43-HIPP1 1–778} in 38D, the DNA of reporter constructs was injected into preblastoderm embryos with the *y*^*1*^*M{vas-int*.Dm}ZH-2Aw*; M{3xP3-RFP.*attP’}ZH-38D* genotype^61^. The resulting flies were crossed with *y*^*2*^*w*^*1118*^ flies, and the progeny carrying the transgene in the 38D region were identified by their eye color. The generation of transgenic lines was performed as previously described^15^. The P{w^+^; UAS-Mod-67.2} construct was described previously^65^.

Three independent *y*^*2*^; *Hipp*^*Δ1*^*mod*(*mdg4*)^*u1*^/*Hipp*^*Δ1*^*mod*(*mdg4*)^*u1*^ lines were generated by genetic recombination. In the initial cross, *y*^*2*^*; Hipp1*^*Δ1*^, *3xPE:dsRed*/*Hipp1*^*Δ1*^, *3xPE:dsRed* females were crossed with *y*^*2*^*; mod(mdg4)*^*u1*^/*mod(mdg4)*^*u1*^ males. Then, the resulting *y*^*2*^*; mod(mdg4)*^*u1*^/*Hipp1*^*Δ1*^, *3xPE:dsRed* females were crossed with *y*^*2*^*; mod(mdg4)*^*u1*^/*mod(mdg4)*^*u1*^ males. In the next generation, *y*^*2*^*; mod(mdg4)*^*u1*^, *Hipp1*^*Δ1*^, *3xPE:dsRed*/*mod(mdg4)*^*u1*^ males were identified by a combination of *dsRed* expression and bristle pigmentation. Selected males were individually crossed with *y*^*2*^*; TM3*, *Sb*/*TM6*, *Hu* females to obtain the stable lines.

To express either *Hipp1* or *Mod(mdg4)-67*.*2* genes on the *Hipp*^*Δ1*^*mod*(*mdg4*)^*u1*^ mutant background, the *y*^*1*^w*; P{Act5C-GAL4-w}E1/*CyO* driver strain was used (Bloomington Center #25374). Obtained *y*^*1*^w*; P{Act5C-GAL4-w}E1/*CyO; mod(mdg4)*^*u1*^, *Hipp1*^*Δ1*^, *3xPE:dsRed*/*TM3*, *Sb* males were crossed with *y*^*2*^*; mod(mdg4)*^*u1*^, *Hipp1*^*Δ1*^, *3xPE:dsRed*/*TM3*, *Sb* females bearing either *P{w*^+^*; UAS-Mod-67*.*2}*/*CyO* or *P{w*^+^*; 5xUAS hsp43-HIPP1 1-778}*/*CyO* on the second chromosome. In progeny, the phenotypes of males without the balancer chromosomes *In(2RL)*, *CyO* and *In(3LR)TM3*, *Sb* were tested.

The effects of various mutation combinations were scored independently, by two researchers. To determine the *yellow* phenotype, the extent of adult pigmentation was visually estimated in 3- to 5-day-old males. For *yellow* phenotypes, wt expression in the abdominal cuticle, wings, and bristles was assigned an arbitrary score of 5, while the absence of *yellow* expression was scored as 1, those flies in which the *yellow* allele was previously characterized as a reference. At least 50 flies were scored.

*Two-hybrid assays, GST pull-down experiments, RNA interference (RNAi) treatment, and immunoprecipitation experiments* were performed as described previously^19^.

### Chromatin preparation and ChIP analysis

Chromatin was prepared from the middle pupa stage^66^, and the resulting chromatin preparation was used for ChIP experiments, as described previously^65^. Immunoprecipitated DNA was quantified using qPCR, with SYBR green (Bio-Rad Cat# 170-8882). Primers were positioned in the middle of the binding region, as identified in ModEncode by ChIP-seq. The primer sequences used in PCR for ChIP analyses are shown in Suppl. Table 1. Analyses were performed on three biological replicates.

### Generation of Hipp1 antibody

Antibodies against HIPP1 (168–332 aa of HIPP1 protein isoform A) were raised in rabbits. The epitope for antibody production was expressed as a 6 × His-tagged fusion protein in *Escherichia coli*, affinity-purified on Ni Sepharose 6 Fast Flow (GE Healthcare), according to the manufacturer’s protocol, and injected into rabbits, following the standard immunization procedure. Antibodies were affinity-purified using the same epitope as was used for immunization. Antibody production was performed according to procedures outlined in the NIH (USA) Guide for the Care and Use of Laboratory Animals. The protocol used was approved by the Committee on Bioethics of the Institute of Gene Biology of the Russian Academy of Sciences. All procedures were performed under conditions designed to minimize suffering.

### Antibodies

Specific antibodies used and their working dilutions were as follows: rat anti-CP190 (1:500), rabbit anti**-**Mod(mdg4)-67.2 (1:500), rat anti-dCTCF (1:300), and rabbit anti Su(Hw) N-terminal domain (1:200) were raised in our lab^65–67^; rabbit antibodies against the C-terminal domain of Su(Hw) (1:200) were kindly provided by M. Erokhin^20^; and affinity-purified rabbit antibody against HIPP1 (1:300).

### Dissection of flies

For each replicate, approximately 100 ovaries from 4- to 6-hour-old virgin females were dissected, as described previously^26,27^.

### RNA isolation and RT-qPCR analysis

For RT-qPCR experiments, total RNA from the heads and ovaries of females was isolated using TRIzol reagent (Invitrogen). Genomic DNA was removed by treatment with DNase I (Fermentas, 1 U per 10 μg), followed by purification with a QIAGEN RNeasy kit. RNA was reverse transcribed into cDNA with a RevertAid H Minus RT Revert Transcriptase kits (Fermentas), following the manufacturer’s instructions. The resulting cDNA was analyzed by qPCR (Bio-Rad CFX 96 Cycler), using SYBR Green. Relative steady-state mRNA levels were determined from the threshold cycle for amplification, using the ΔΔCT method. Each experiment was performed in two to three independent biological replicates, and the results were averaged. The expression levels of each gene were determined using *ras64B* as an internal control. Primer sequences used for RT-PCR analyses are listed in Suppl. Table 2.

## Supplementary information

Supplementary Materials

## Data Availability

All data generated or analyzed during this study are included in this published article and its Supplementary Information file.
