# Supplementary material for: HIPP1 stabilizes the interaction between CP190 and Su(Hw) in the Drosophila insulator complex
Source: Sci Rep. 2019 Dec 13;9:19102. doi: 10.1038/s41598-019-55617-6 (PMC6911044; doi:10.1038/s41598-019-55617-6)
Supplement: Supplementary file 1 — Supplementary Materials [file 41598_2019_55617_MOESM1_ESM.pdf]

HIPP1 stabilizes the interaction between CP190 and Su(Hw) in the *Drosophila* insulator complex

Larisa Melnikova[a], Varvara Molodina[a], Maksim Erokhin[b], Pavel Georgiev[b] and Anton Golovnin[a,]*

[a]*Department of Drosophila Molecular Genetics, Institute of Gene Biology, Russian Academy of Sciences, 34/5 Vavilov St., 119334 Moscow, Russia*

[b]*Department of the Control of Genetic Processes, Institute of Gene Biology, Russian Academy of Sciences, 34/5 Vavilov St., 119334 Moscow, Russia*

**Corresponding author:*

Anton Golovnin[1]. E-mail: agolovnin@mail.ru

Supplementary Figures

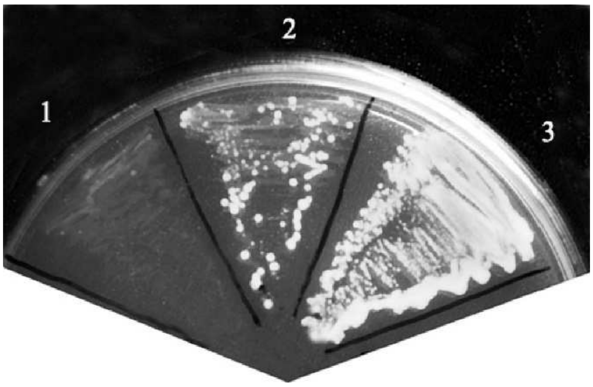

Suppl. Fig. 1. Representative examples of colony growth on selective medium in yeast two-hybrid assay.

(1) No colony growth (designated "–") indicative of the absence of interaction in negative control with the empty prey vector pGAD expressing the GAL4 activation domain alone and the bait vector pGBT expressing a test protein fused with the GAL4 DNA-binding domain. None of the test proteins showed self-activation in control experiments. (2) Moderate colony growth (designated "+") and (3) abundant colony growth (designated "++") indicative of intermediate and strong interaction between the test proteins, respectively.

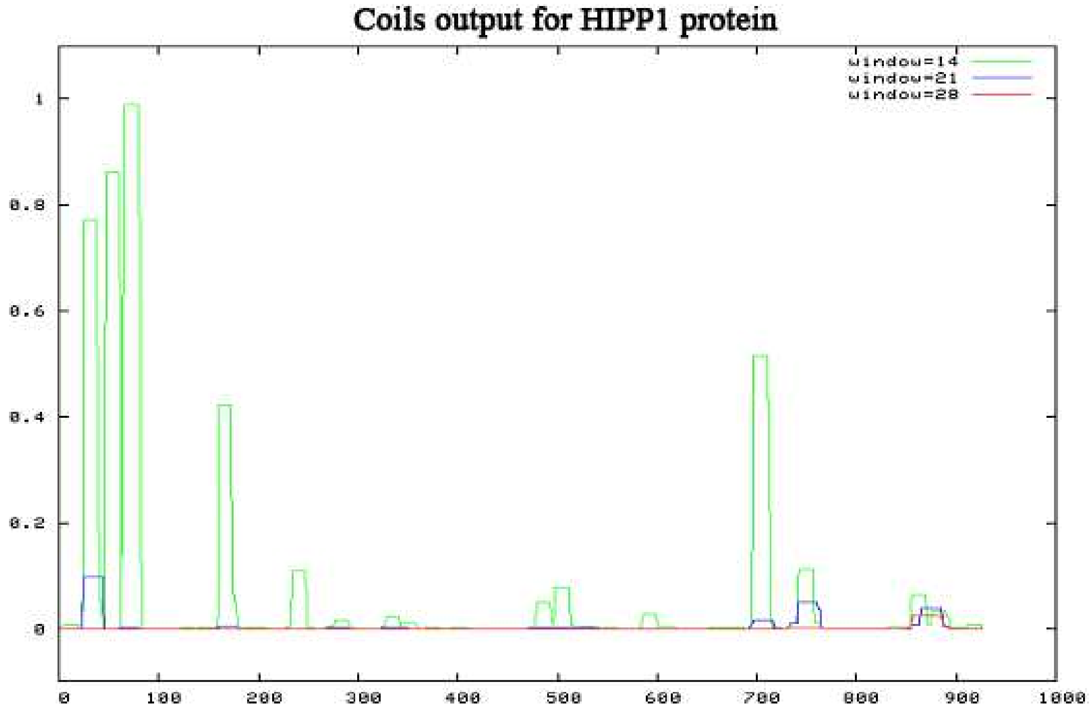

Suppl. Fig. 2. Coiled coil regions in the HIPP1 protein predicted from its sequence using the ExPASy COILS tool [1].

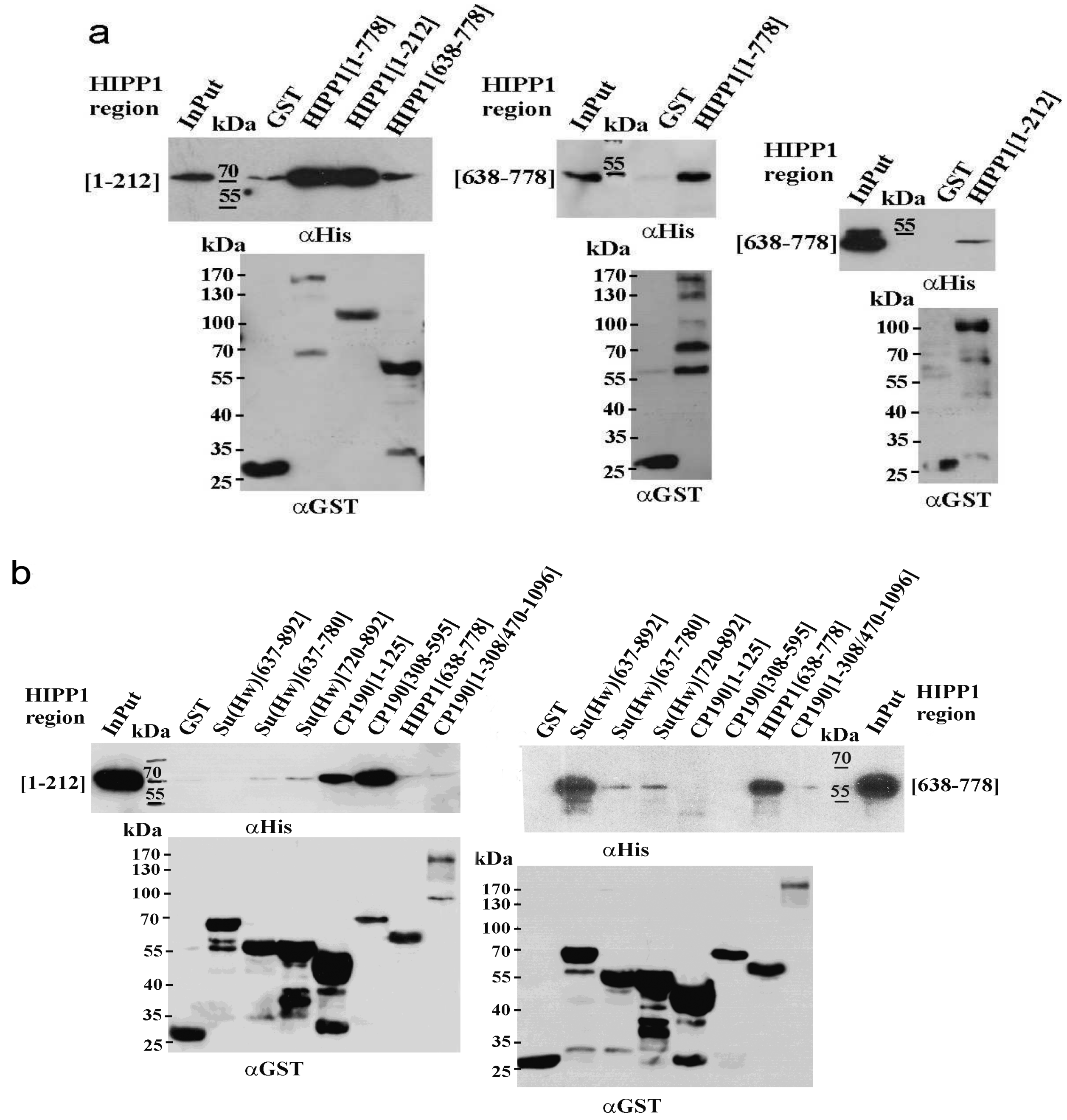

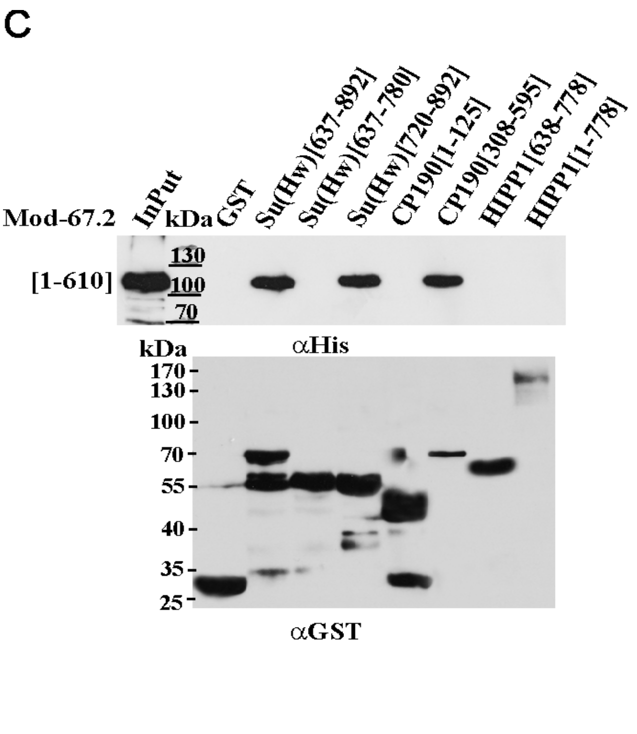

Suppl. Fig. 3. Analysis of interactions between HIPP1 and insulator proteins in GST pull-down assay.

(a) Test for direct interaction between GST-fusion and His-fusion HIPP1 regions.

(b) Test for direct interaction between GST-fusion Su(Hw) and CP190 derivatives and His-fusion HIPP1 regions.

(c) Test for direct interaction between GST-fusion Su(Hw), CP190, and HIPP1 derivatives and His-fusion full-length Mod(mdg4)-67.2 protein.

For GST pull-down experiments, GST-fused proteins (HIPP1$^{1-778}$, HIPP1$^{1-212}$, HIPP1$^{638-778}$, Su(Hw)$^{637-892}$, Su(Hw)$^{637-780}$, Su(Hw)$^{720-892}$, CP190$^{1-125}$, CP190$^{308-595}$, CP190$^{1-308/470-1096}$), or GST alone was incubated with Glutathione Sepharose 4B beads in binding buffer (20 mM Hepes-KOH, pH 7.6; 200 mM KCl, 2.5 mM $MgCl_2$, 10% glycerol, 0.05% NP40) for 2 h. The beads were then blocked in 5% BSA for 1 h and incubated in pairs with 6His-tagged HIPP1 derivatives (HIPP1$^{1-212}$, HIPP1$^{638-778}$) or full-length Mod(mdg4)-67.2 (Mod-67.2 $^{1-610}$) for 3 h. After incubation, the beads were washed three times in wash buffer (10 mM Tris-HCl, pH 7.5; 1 mM EDTA, 0.2% NP40, 300 mM NaCl), boiled in Laemmli buffer, and resolved in 8% SDS PAAG. The proteins were blotted onto a PVDF membrane, which was then consecutively probed with antibodies to His (Amersham, designated αHis) or to GST (Amersham, designated αGST) used as loading control (at the bottom). The sample in the InPut lane contained 10% of His-fusion protein loaded onto Glutathione Sepharose together with GST-fusion proteins. The sample in the GST lane contained GST alone used as negative control. Numbers in brackets refer to amino acid residues. All results were reproduced in three independent experiments.

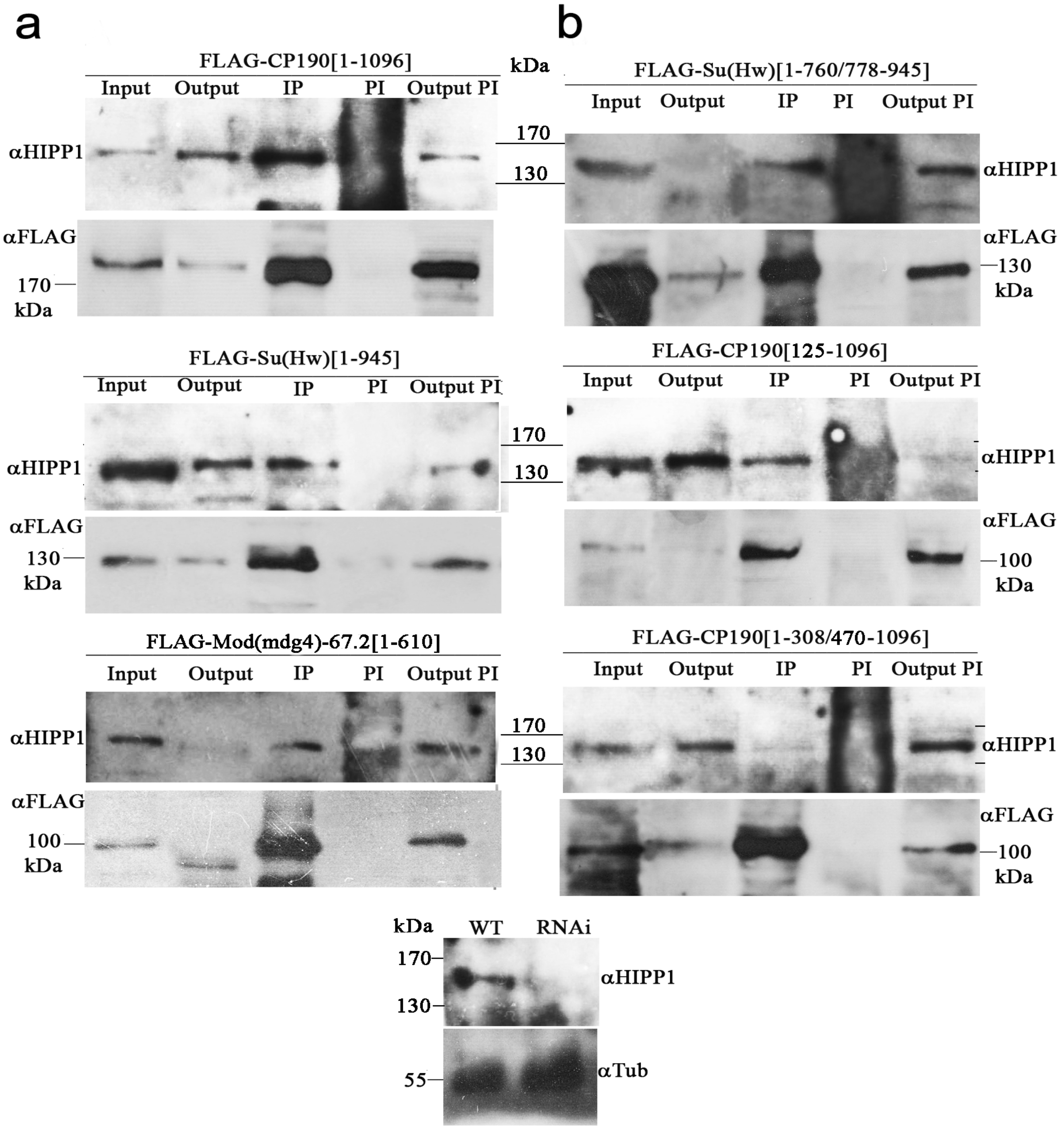

Suppl. Fig. 4. Results of co-immunoprecipitation between endogenous HIPP1 and FLAG-tagged insulator proteins in S2 cells.

(a) Test for interaction between HIPP1 and full-length CP190, Su(Hw), and Mod(mdg4)-67.2 proteins.

(b) Test for interaction between HIPP1 and CP190, Su(Hw), and Mod(mdg4)-67.2 derivatives.

The immunoprecipitated complexes were washed with 150 mM KCl-containing buffers before loading onto SDS-PAGE for Western blot analysis. The PVDF membrane was consecutively probed with antibodies against the HIPP1 protein (designated αHIPP1) or FLAG epitope to visualize immunoprecipitated target proteins. Input is the input fraction (10% of lysate used for immunoprecipitation); Output, the supernatant after immunoprecipitation; IP, the

immunoprecipitate; PI, immunoprecipitation with nonspecific IgG; Output PI, the supernatant after nonspecific IgG immunoprecipitation. The result of test for the specificity of HIPP1 antibodies by RNAi method is shown on the bottom panel. Anti-tubulin staining (αTub) was used as loading control. All experiments were repeated in triplicate.

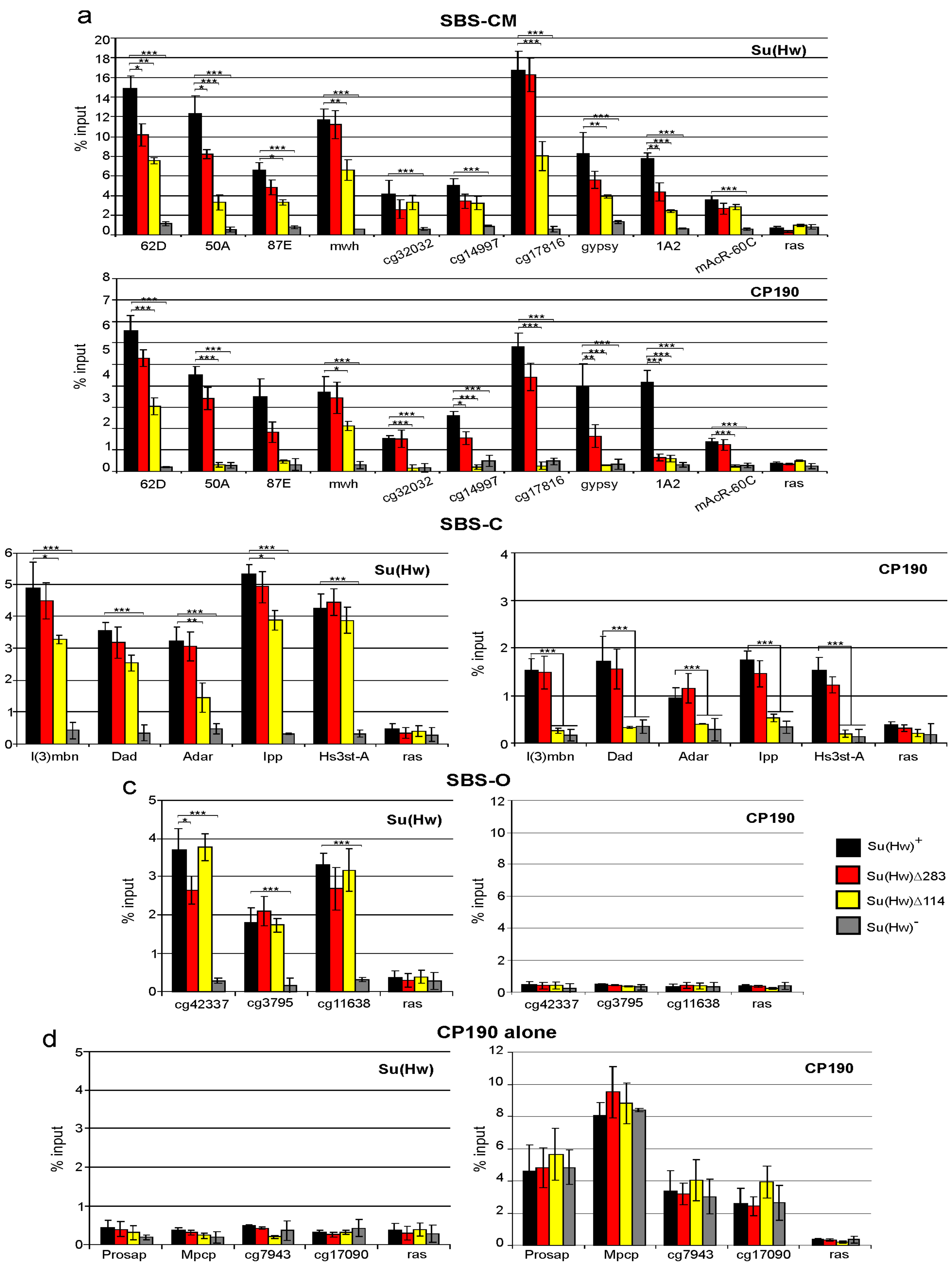

Suppl. Fig. 5. Binding of Su(Hw) and CP190 proteins to genomic sites in pupae from transgenic lines expressing full-length Su(Hw)$^{+}$, Su(Hw)$^{\Delta 283}$, and Su(Hw)$^{\Delta 114}$.

(a) SBS-CM sites; (b) SBS-C sites; (c) SBS-O sites; (d) CP190 alone sites. Su(Hw)$^{-}$ refers to the *y*^{2} *w*^{1118}; *su(Hw)*^{v}*/su(Hw)*^{e04061} background.

The different variants of the Su(Hw) protein were expressed in the *y*^{2}*sc*^{D1}*ct*^{6}; *P{Su(Hw)}-38D/P{Su(Hw)}-38D*; *su(Hw)*^{v}*/su(Hw)*^{e04061} lines, where P{Su(Hw)} are
Su(Hw)$^{+}$ – *P{w*$^{+}$*;UbqW-Su(Hw)1-945-FLAG}/ P{w*$^{+}$*;UbqW-Su(Hw)1-945-FLAG};*
Su(Hw)Δ114 – *P{w*$^{+}$*;UbqW-Su(Hw)1-88/202-945-FLAG}/ P{w*$^{+}$*;UbqW- Su(Hw)1-88/202-945-FLAG};*
Su(Hw)Δ283 – *P{w*$^{+}$*;UbqW-Su(Hw)1-760/778-945-FLAG}/ P{w*$^{+}$*;UbqW- Su(Hw) 1-760/778-945-FLAG}.*
Primers were positioned in the middle of the binding region identified in ModEncode by ChIP-seq. PCR products were amplified from two separate immunoprecipitates of three different chromatin preparations. The *ras64B* coding region (ras) was used as a control devoid of Su(Hw) binding sites. The percent recovery of immunoprecipitated DNA (Y axis) was calculated relative to the amount of input DNA. Error bars indicate standard deviation of three independent biological replicates. Asterisks indicate significance levels of *$p < 0.05$, **$p < 0.01$, or ***$p < 0.001$. P values are not displayed for significance >0.05 (here and in Supl. Fig. 7). ChIP was performed with antibodies against the Su(Hw) C-terminal region, and CP190. Statistical analysis (Student's *t*-test) was performed relative to the Su(Hw)+ variant. Significance levels are shown for treatment comparisons indicated by horizontal brackets.

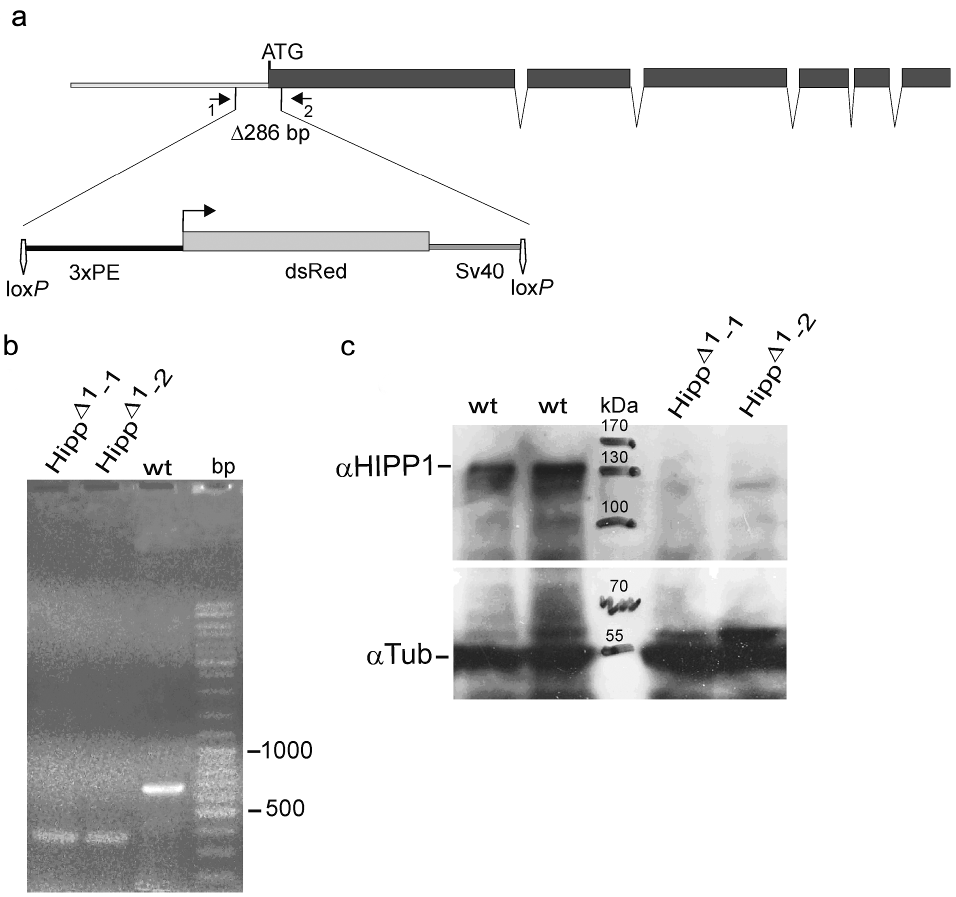

Suppl. Fig. 6. A null mutation generated in the gene encoding HIPP1 using the genome editing CRISPR/Cas9 approach.

(a) Scheme (not to scale) of the *Hipp1* gene with exons gray rectangles) and introns between them. A 286-bp sequence including the transcription start site and ATG codon was deleted (Δ286 bp) and substituted by the *loxP*-flanked *dsRed* reporter under control of the 3xPE promoter. SV40 is transcription terminator from SV40 virus. Arrows show the positions and directions of primers 1 and 2 used in PCR.

(b) PCR analysis of flies homozygous for $Hipp^{\Delta 1}$. $HIPP1^{\Delta 1}$-1 and $HIPP1^{\Delta 1}$-2 are lines with the null mutation in the *Hipp1* gene; wt, wild type line. PCR was performed after deleting the *3xPE–dsRed–SV40* sequence by Cre-mediated recombination. The size of the bands in the mutant lines is smaller than in the wild type line, which confirms the presence of the deletion.

(c) Western blot analysis of flies homozygous for $Hipp^{\Delta 1}$. HIPP1 isolated from adult flies as described [2] was resolved by electrophoresis in 7.5% SDS-PAAG, electroblotted onto a PVDF membrane, and probed with antibodies against HIPP1 (designated αHIPP1). Anti-tubulin staining (αTub) was used as loading control. Lines included in analysis are indicated above the figure. The absence of the bands in the mutant lines, compared to the wild type line, confirms the presence of the deletion.

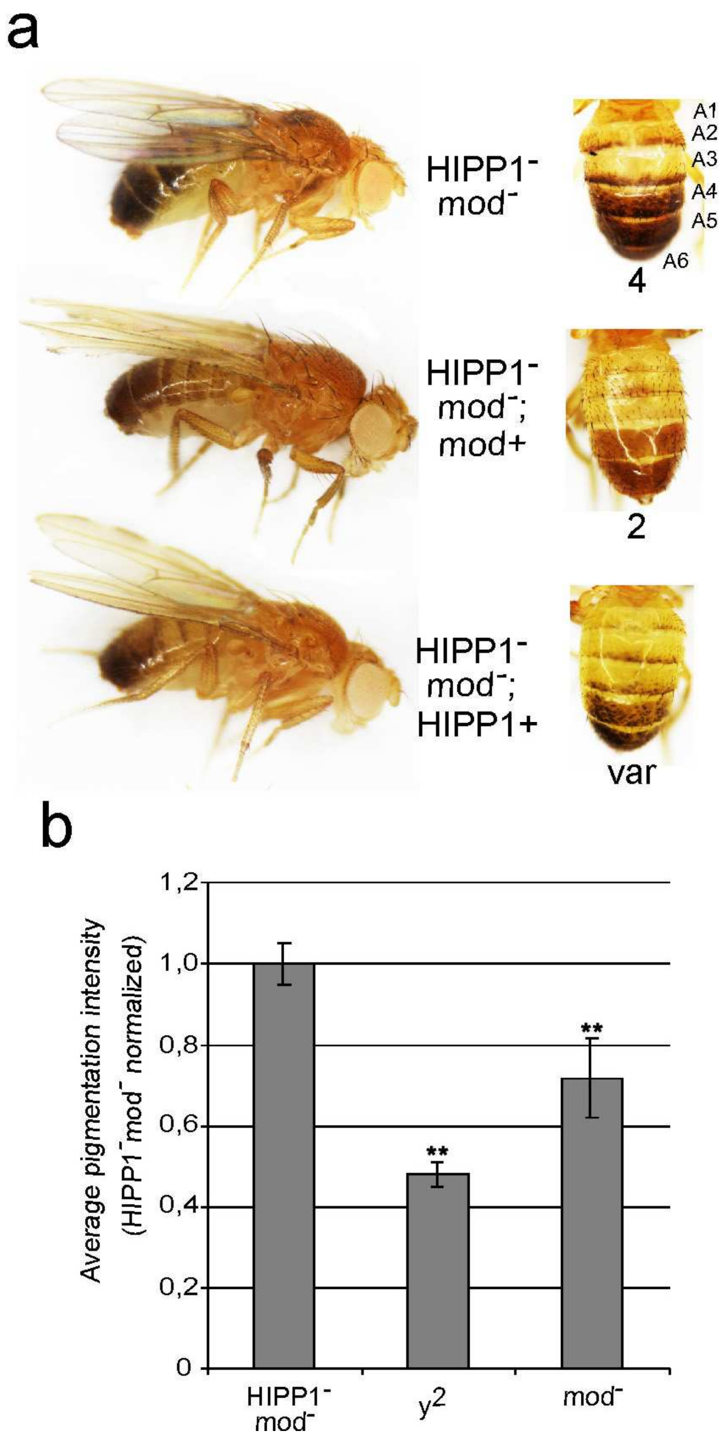

Suppl. Fig. 7. Effects of the *Hipp1+* or *Mod(mdg4)-67.2+* transgenes on *gypsy* enhancer-blocking activity in the y^2; $Hipp^{\Delta 1}mod(mdg4)^{u1}/Hipp^{\Delta 1}mod(mdg4)^{u1}$ line.

(a) Images show the effects of the *P{w⁺;UAS-Mod-67.2}* – [mod+], and *P{w⁺; 5xUAS hsp43-HIPP1 1-778}* – [HIPP1+] transgenes on the *yellow* phenotype and on abdomen pigmentation in three-day-old. y^2;$Hipp^{\Delta 1}mod(mdg4)^{u1}/Hipp^{\Delta 1}mod(mdg4)^{u1}$ – [HIPP1⁻mod⁻] males. Numbers show the *yellow* expression scores in the abdominal segments (4, moderate pigmentation; 2, weak pigmentation; var, variegated pigmentation).

(b) Quantitative assessment of the pigmentation intensity of the abdominal A5 segments. Images were analysed using the Measure tool in Fiji. “Mean” parameters were used for histogram generation. For each genotype, 20 representative images were processed. Pigmentation intensity (Y axis) is shown as fold change relative to the HIPP1⁻mod⁻ variant. Error bars indicate standard deviation of pigmentation intensity. Asterisks indicate significance levels (Student's *t*-test) of $**p < 0.01$. Statistical analysis was performed relative to the HIPP1⁻mod⁻ variant.

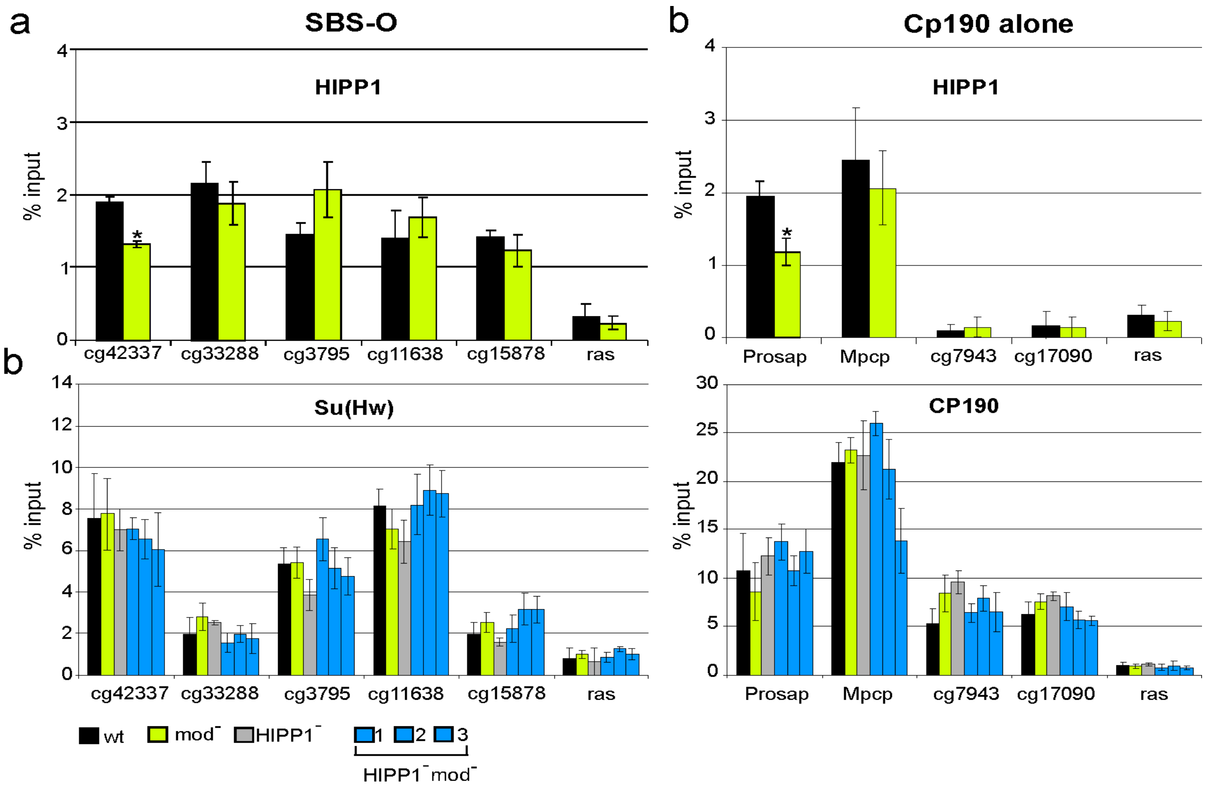

Suppl. Fig. 8. Binding of Su(Hw) and CP190 proteins to genomic sites in pupae from lines bearing *mod(mdg4)^{u1}* and *Hipp$^{\Delta 1}$* mutations.

(a) The HIPP1 and Su(Hw) binding to the SBS-O sites; (b) the HIPP1 and CP190 binding to the CP190 alone sites. The lines used in analysis are designated as [wt] – y^2 w^{1118}; [mod $^-$] – y^2 w^{1118}, *mod*(*mdg4*)^{u1}/ *mod*(*mdg4*)^{u1}, [HIPP $^-$] – y^2 w^{1118}; *Hipp*$^{\Delta 1}$/ *Hipp*$^{\Delta 1}$, and [HIPP $^-$ mod $^-$] – y^2 w^{1118}; *Hipp*$^{\Delta 1}$ *mod*(*mdg4*)^{u1}/ *Hipp*$^{\Delta 1}$ *mod*(*mdg4*)^{u1}. ChIP was performed with antibodies against the Su(Hw) N-terminal domain, HIPP1, and CP190. Statistical analysis was performed relative to wild type condition. Other designations as in Suppl. Fig.5.

Supplementary Materials

Constructs for yeast two-hybrid assay

Constructs **pGBT9Su(Hw)** and **pGDASu(Hw)** containing full-length Su(Hw) 1–945, **pGBT9Su(Hw)$^{\Delta C}$** and **pGDASu(Hw)$^{\Delta C}$** containing Su(Hw) 1–892, **pGBT9Su(Hw)$^{1\text{-}720}$**, and **pGDASu(Hw)$^{1\text{-}720}$** were described previously [3,4].

To generate **pGBT9Su(Hw)$^{1\text{-}801}$** plasmid, Su(Hw) cDNA was PCR-amplified from pGEM3ZfSu(Hw) with primers 5'-aatgagtgcctccaaggagggc-3' (upstream) and 5'-agtcgacctttgcagagcaccgtctcct-3' (downstream, containing *Sal*I site). The PCR product was digested with *Sal*I and cloned in the pGBT9 vector cleaved with *Sma*I and *Sal*I.

To generate **pGDASu(Hw)$^{1\text{-}801}$** plasmid, cDNA of Su(Hw) was PCR-amplified from pGEM3ZfSu(Hw) with primers 5'-aatgagtgcctccaaggagggc-3' (upstream) and 5'-aaccatggctttgcagagcaccgtctcct-3' (downstream, containing *Nco*I site). This PCR product was digested with *Nco*I and cloned in pGDA cleaved with *Hpa*I and *Nco*I.

To generate **pGBT9Su(Hw)$^{637\text{-}892}$** plasmid, Su(Hw) cDNA was PCR-amplified from pGBTSu(Hw)ΔC with upstream primer 5'- aatggttaaacacatcagcgggctc-3' (with introduced ATG codon) and downstream plasmid primer. The PCR product was digested with *Sal*I and cloned in the pGBT9 vector cleaved with *Sma*I and *Sal*I.

To generate **pGDASu(Hw)$^{637\text{-}892}$** plasmid, cDNA of Su(Hw) was PCR-amplified from pGBTSu(Hw)ΔC with primers 5'- aatggttaaacacatcagcgggctc-3' (upstream, with introduced ATG codon) and 5'-tccatggtttcgcctgtgaccgaca-3' (downstream, containing *Nco*I site). This PCR product was digested with *Nco*I and cloned in pGDA cleaved with *Hpa*I and *Nco*I.

To generate **pGBT9Su(Hw)$^{637\text{-}780}$** plasmid, Su(Hw) cDNA was PCR-amplified from pGBTSu(Hw)ΔC with upstream primer 5'-aatggttaaacacatcagcgggctc-3' (with introduced ATG codon) and downstream plasmid primer. The PCR product was digested with *Bam*HI and cloned in the pGBT9 vector cleaved with *Sma*I and *Bam*HI.

To generate **pGDASu(Hw)$^{637\text{-}780}$** plasmid, cDNA of Su(Hw) was PCR-amplified from pGBTSu(Hw)ΔC with upstream primer 5'-aatggttaaacacatcagcgggctc-3' (with introduced ATG codon) and downstream plasmid primer. This PCR product was digested with *Bam*HI and cloned in pGDA cleaved with *Hpa*I and *Bam*HI.

To generate **pGBT9Su(Hw)$^{720\text{-}892}$** plasmid, Su(Hw) cDNA was PCR-amplified from pGBTSu(Hw)ΔC with upstream primer 5'-atgaggcgcaggtctcgccggt-3' (with introduced ATG codon) and downstream plasmid primer. The PCR product was digested with *Bam*HI and cloned in the pGBT9 vector cleaved with *Sma*I and *Sal*I.

To generate **pGDASu(Hw)$^{720-892}$** plasmid, cDNA of Su(Hw) was PCR-amplified from pGBTSu(Hw)ΔC with primers 5'-atgaggcgcaggtctcgccggt-3' (upstream, with introduced ATG codon) and 5'-tccatggtttcgcctgtgaccgaca-3' (downstream, containing *Nco*I site). The PCR product was digested with *Nco*I and cloned in pGDA cleaved with *Hpa*I and *Nco*I.

To generate **pGBT9Su(Hw)$^{1-637}$** plasmid, Su(Hw) cDNA was PCR-amplified from pGBTSu(Hw)ΔC with upstream plasmid primer and downstream 5'-agtcgactcctccgattcgatgtccttc-3' primer containing *Sal*I site. The PCR product was digested with *Eco*RI and *Sal*I and cloned in the pGBT9 vector cleaved with the same enzymes.

To generate **pGDASu(Hw)$^{1-637}$** plasmid, cDNA of Su(Hw) was PCR-amplified from pGBTSu(Hw)ΔC with primers 5'-aatgagtgcctccaaggagggc-3' and 5'-tccatggtcctccgattcgatgtccttc-3' (downstream, containing *Nco*I site). This PCR product was digested with *Nco*I and cloned in pGDA cleaved with *Hpa*I and *Nco*I.

To generate **pGBT9Su(Hw)1-760/778-945** plasmid, the fragment containing the corresponding deletion was amplified from genomic DNA of Su(Hw)Δ283 transgenic line, digested with *Bam*HI and *Eco*RI, and ligated into pGBTSu(Hw)ΔC cleaved with the same enzymes. The 2019-bp *Eco*RI–*Eco*RI region of Su(Hw) cDNA was then reintroduced into the *Eco*RI site of the resulting plasmid and checked for orientation.

To generate **pGDASu(Hw)$^{1-760/778-945}$** plasmid, the *Xba*I–*Sal*I fragment from pGBT9Su(Hw) 1-760/778-945 plasmid was subcloned in pGDASu(Hw)ΔC cleaved with the same enzymes.

Plasmids **pGBT9CP190$^{1-1096}$** and **pGDA190$^{1-1096}$**, **pGBT9CP190$^{1-765}$** and **pGDA190$^{1-765}$**, **pGBT9CP190$^{1-125}$** and **pGDA190$^{1-125}$**, **pGBT9CP190$^{125-1096}$** and **pGDA190$^{125-1096}$**, **pGBT9CP190$^{1-595}$** and **pGDA CP190$^{1-595}$**, **pGBT9CP190$^{1-308}$** and **pGDA190$^{1-308}$** were described previously [3,4].

To generate **pGBT9CP190$^{308-470}$** and **pGDACP190$^{308-470}$,** the fragment *Eco*RI–*Apa*I (filled in with Klenow) from CP190 cDNA was subcloned into pGBT cleaved with *Eco*RI and *Sal*I (filled in with Klenow fragment). Then the *Eco*RI–*Sal*I fragment from the resulting plasmid was subcloned into pGDA cleaved with the same enzymes.

To generate **pGBTCP190$^{308-595}$** and **pGDACP190$^{308-595}$**, plasmids pGBT9CP190$^{1-595}$ and pGDACP190$^{1-595}$ were cleaved with *Eco*RI and then religated without the 1-308 N-terminal fragment.

To generate **pGBT9HIPP1$^{1-778}$** and **pGDAHIPP1$^{1-778}$** plasmids, HIPP1 cDNA was amplified with primers 5’-gaattcatggagcaggtgtcggata-3’ (with introduced *Eco*RI site) and 5’-ggatccaaaatcaacgtcgttaagatagc-3’ (with introduced *Bam*HI site) from cDNA library. The PCR product was cleaved with *Bam*HI and *Eco*RI, and the 638-bp *Bam*HI–*Eco*RI fragment was

ligated into either pGBT9 or pGDA vector cleaved with the same enzymes. The remaining 2100-bp *Eco*RI–*Eco*RI fragment of cDNA was then cloned into the *Eco*RI site of the resulting plasmid and checked for orientation.

To generate **pGBT9HIPP1**$^{\mathbf{1\text{-}212}}$ and **pGDAHIPP1**$^{\mathbf{1\text{-}212}}$ plasmids, the *Eco*RI–*Kpn*I fragment (filled in with Klenow fragment) from pGBT9HIPP1$^{1\text{-}778}$ was cloned into pGBT9 cleaved with *Eco*RI and *Sma*I or into pGDA cleaved with *Eco*RI and *Bam*HI (filled in with Klenow fragment).

To generate **pGBT9HIPP1**$^{\mathbf{638\text{-}778}}$ and **pGADHIPP1**$^{\mathbf{638\text{-}778}}$ plasmids, the *Bgl*I–*Bam*HII fragment (filled in with Klenow fragment) from pGBT9HIPP1$^{1\text{-}778}$ was cloned into pGBT cleaved with *Eco*RI (filled in with Klenow fragment) and *Bam*HI or into pGAD cleaved with *Eco*RI (filled in with Klenow fragment) and *Bam*HI.

To generate **pGBT9HIPP1**$^{\mathbf{212\text{-}638}}$ and **pGDAHIPP1**$^{\mathbf{212\text{-}638}}$ plasmids, HIPP1 cDNA was amplified from pGBT9HIPP1$^{1\text{-}778}$ plasmid with primers 5’-gaattcatggtgatgggcttggacgcaggta-3’ (with introduced *Eco*RI site and ATG codon) and 5’-ggatcccctttggctcgacttgcttac-3’ (with introduced *Bam*HI site) primers. The PCR product was cleaved with *Eco*RI and *Bam*HI and subcloned either in pGBT9 or in the pGDA plasmids digested with the same enzymes.

Constructs for GST Pull Down assay

First, the pSKHIPP1 plasmid was generated. The pGBT9HIPP1$^{1\text{-}778}$ plasmid was cleaved with *Bam*HI and *Eco*RI; the 638-bp *Bam*HI–*Eco*RI fragment was ligated into pBluescript II SK(+) cleaved with the same enzymes, and the remaining 2100-bp *Eco*RI–*Eco*RI fragment of cDNA was then cloned into the *Eco*RI site of the resulting plasmid and checked for orientation.

pGex4T-1 HIPP1$^{\mathbf{1\text{-}778}}$ was generated by cloning the *Xho*I–*Not*I fragment from pSKHIPP1 into pGex4T-1 cleaved with same enzymes.

pGex4T-1 HIPP1$^{\mathbf{638\text{-}778}}$ was generated by cloning the BglI–NotI fragment (filled in with Klenow fragment) from pSKHIPP1 into pGex4T-1 cleaved with *Eco*RI (filled in with Klenow fragment) and *Not*I.

pGex4T-1 HIPP1$^{\mathbf{1\text{-}212}}$ was generated by cloning the *Eco*RI–*Eco*RI fragment from pGBT9HIPP1$^{1\text{-}212}$ into pGex4T-1 cleaved with *Eco*RI. Insert orientation was checked by PCR.

pET32aSmaHIPP1$^{\mathbf{638\text{-}778}}$ was generated by cloning the *Bgl*I–*Not*I fragment (filled in with Klenow fragment) from pSKHIPP1 into pET32aSma cleaved with *Eco*RV and *Not*I.

pET32aSmaHIPP1$^{\mathbf{1\text{-}212}}$ was generated by cloning the *Eco*RI–*Eco*RI fragment (filled in with Klenow fragment) from pGBT9HIPP1$^{1\text{-}212}$ into pET32aSma cleaved with *Bam*HI (fill in with Klenow fragment) and *Xho*I (filled in with Klenow fragment). Insert orientation was checked by PCR.

pGex4T-1Su(Hw)$^{637-892}$ and **pGex4T-1Su(Hw)**$^{637-780}$ was generated by cloning the *Hinc*II–*Hinc*II fragment from pGBT9Su(Hw)$^{637-892}$ or pGBT9Su(Hw)$^{637-780}$ into pGex4T-1 cleaved with *Sma*I enzyme, checking insert orientation.

pGex4T-1Su(Hw)$^{720-892}$ was generated by cloning the *Eco*RI–*Sal*I fragment from pGBT9Su(Hw)$^{720-892}$ into pGex4T-1 cleaved with the same enzymes.

pGex4T-1CP190$^{308-595}$ was generated by cloning the 891-bp *Bam*HI–*Eco*RI fragment from CP190 cDNA into pGex4T-1 cleaved with the same enzymes.

pGex4T-1CP190$^{1-308/\ 420-1096}$ was generated by cleaving CP190 cDNA with *NheI* and *BamHI,* than fill in with Klenow fragment and relegate. The 1911-bp SalI–SalI fragmen wich containing deletion was ligated into pGex4T-1 CP190 cleaved with the same enzymes and checked for orientation

pGex4T-1CP190$^{1-125}$ was described previously [4].

Constructs for transient transfection of S2 cell culture.

Full-length FLAG-tagged Su(Hw)$^{1-945}$ expressed in pAc5.1 was described previously [5].

To prepare **pAc5.1CP190**$^{1-1096}$**-FLAG** plasmid, the *Hind*III–*Bst*XI fragment containing full-length CP190 cDNA was cloned in frame with FLAG epitope into pAc5.1 cleaved with the same enzymes.

To prepare **pAc5.1Mod(mdg4)-67.2**$^{1-610}$**-FLAG** plasmid, the *Eco*RI–*Bam*HI fragment containing full-length Mod(mdg4) cDNA was cloned in frame with FLAG epitope into pAc5.1 cleaved with the same enzymes.

To prepare **pAc5.1CP190**$^{1-308/470-1096}$**-FLAG**, the *Bam*HI–*Xba*I fragment from pGBT9CP190$^{1-308/470-1096}$ was cloned into the corresponding vector at *Bam*HI and *Xba*I sites.

To generate **pAc5.1CP190**$^{125-1096}$**-FLAG** plasmid, the *Nco*I–*Eco*RV promoter region fragment (filled in with Klenow fragment) from pAc5.1 was cloned into pAc5.1CP190$^{1-1096}$-FLAG cleaved with *Nco*I and checked for orientation.

To prepare **pAc5.1Su(Hw)**$^{1-760/778-945}$**-FLAG,** the *Hinc*II–*Hind*III fragment from pGBT9 Su(Hw)$^{1-760/778-945}$ was subcloned into pAc5.1Su(Hw)$^{1-945}$ cleaved with *Xho*I (filled in with Klenow fragment) and *Hind*III.

Transgenic constructs

Constructs **P{w$^+$;UbqW-Su(Hw)1-954-FLAG}, P{w$^+$;UbqW-Su(Hw)Δ114-FLAG},** and **P{w$^+$;UbqW-Su(Hw)1-760/778-945-FLAG}** were described previously [3,4,6].

To generate **P{w+; 5xUAS hsp43-HIPP1 1-778}** cDNA of HIPP1 digested by XhoI-SpeI from

pGBT9HIPP1$^{1-778}$ plasmid was cloned in pUAST-B vector digested by XhoI and XbaI enzymes.

CRISPR/Cas9 genome editing

HIPP1$^{\Delta 1}$ mutants were generated by the homology-directed repair (HDR) method. For gRNA expression, the U6:3 promoter was used [7], and target sites were designed so that they directed Cas9-mediated cleavage to the 5' end of the coding sequence, which targets the HIPP1 promoter: 5'-cctatcgatagatccacctgcgc-3' and 5'-gactctagaactaaaaacggcgg-3'. To reduce the risk of off-target cleavage, target sites were chosen so as to have no highly homologous sites elsewhere in the genome. Off-target potential was assessed using CRISPR target finder Crispr fly design (http://www.crisprflydesign.org). Because a 5′ guanine is required for transcription from U6 promoters, target sites lacking this feature were extended in the 5′ direction by a single guanine. pHD-DsRed-attP was used as a vector to generate dsDNA donor templates for HDR. This vector is designed for replacing a targeted locus with a 50-bp attP phage recombination site and is positively marked with a Cre recombinase-removable 3XP3-dsRed construct for screening. It has two multiple cloning sites (MCS) for inserting homology arms that immediately flank the targeted locus. The 5′ and 3′ homology arms were PCR-amplified from nos-Cas9 flies genomic DNA using primers 5'-acatggcaagccgtcgcttgat-3' and 5'-cgtgaaaatatcggaaaaagtggat-3' for the 5' arm and 5'-cgttagaaacactctcggaaag-3' and 5'-ggtgttggttccacagcaact-3' for the 3' arm. Two vectors expressing gRNAs and template for HDR vector were simultaneously injected into preblastoderm embryos.

References

1 Lupas, A., Van Dyke, M. & Stock, J. Predicting coiled coils from protein sequences. *Science* **252**, 1162-1164, doi:10.1126/science.252.5009.1162 (1991).

2 Gdula, D. A. & Corces, V. G. Characterization of functional domains of the su(Hw) protein that mediate the silencing effect of mod(mdg4) mutations. *Genetics* **145**, 153-161 (1997).

3 Melnikova, L. *et al.* Multiple interactions are involved in a highly specific association of the Mod(mdg4)-67.2 isoform with the Su(Hw) sites in Drosophila. *Open Biol* **7**, doi:10.1098/rsob.170150 (2017).

4 Melnikova, L. *et al.* Interactions between BTB domain of CP190 and two adjacent regions in Su(Hw) are required for the insulator complex formation. *Chromosoma* **127**, 59-71, doi:10.1007/s00412-017-0645-6 (2018).

5 Melnikova, L., Kostyuchenko, M., Parshikov, A., Georgiev, P. & Golovnin, A. Role of Su(Hw) zinc finger 10 and interaction with CP190 and Mod(mdg4) proteins in recruiting the Su(Hw) complex to chromatin sites in Drosophila. *PLoS One* **13**, e0193497, doi:10.1371/journal.pone.0193497 (2018).

6 Melnikova, L. *et al.* The same domain of Su(Hw) is required for enhancer blocking and direct promoter repression. *Sci Rep* **9**: 5314, doi: 10.1038/S41598-019-41761-6 (2019).

7 Port, F., Chen, H. M., Lee, T. & Bullock, S. L. Optimized CRISPR/Cas tools for efficient germline and somatic genome engineering in Drosophila. *Proc Natl Acad Sci U S A.* **111**, E2967-2976, doi:10.1073/pnas.1405500111 (2014).

Supplementary Table 1.

Primer sequences used in ChIP-qPCR analysis

Primer pair	Sequences
mAcR-60C TS2 d<br>mAcR-60C TS2 r	5'- CGCTCTCTCTCGGCTGCT -3'<br>5'- CGCGTTTGTTTTTCACTCGGTTA-
mwh d<br>mwh r	5' -CGATGGTTGACAGGTGAGCAA- 3'<br>5' -TGGTTGCTAAGAACTTCGTTCG- 3'
l(3)mbn d<br>l(3)mbn r	5' -GAGACGCATCACGAAGATACTG- 3'<br>5' -CTCCCAAATGCATAGTCGGCT- 3'
cg32032 d<br>cg32032 r	5' -ATCTCCACCCGCTCATTGGAA- 3'<br>5' -TCAGACAGAGGCAAGTGGCAT- 3'
cg17816 d<br>cg17816 r	5' -CCTTGTTCCTGCGATGATCTCA- 3'<br>5' -CAGAAGTGCGATATGCAGGCA- 3'
cg14997 d<br>cg14997 r	5' -GTGTAATGGCCAGTTACCAGC- 3'<br>5' -TGTGCTCGGACCACTTGCTT- 3'
62D d<br>62D r	5' TTTGGGCTTGGTGAGAACAG 3'<br>5' TGATACCAGGCGAACAGAAATC 3'
50A d<br>50A r	5' ATACAAAGTGGTTTCAGCCAAGAAG 3'<br>5' TTGATAAATAGTCCAGCACGCATAC 3'
87E d<br>87E r	5' GGATGTTACA TTGAGAGTGCTTAGG 3'<br>5' TTTGCGTTTCGGCTGCTGTC 3'
1A2 d<br>1A2 r	5' ACCACACATCAGTCATCGTGT 3'<br>5' CTTCGTCTACCGTTGTGC 3'
gypsy d<br>gypsy r	5' TTCTCTAAAAAGTATGCAGCACTT 3'<br>5' CACGTAATAAGTGTGCGTTGA 3'
cg42337 d<br>cg42337 r	5' TTGATGTCCCTCCGCTCTCA 3'<br>5' CCAACATTGACCATCCAACGAG 3'
cg15878 d<br>cg15878 r	5' GTGAACTCAATTTGCTGGCATC 3'<br>5' CGTTGTGTTCGCCGCTTTTG 3'
cg3795 d<br>cg3795 r	5' CTCCACTGCTGTCAGCATATG 3'<br>5' ATGTCTAGCTGGTCCCTGTTC 3'
cg11638 d<br>cg11638 r	5' TCTCTCTGCCACACTGGACA 3'<br>5' TCATATTCGGCACAGTAAGAGTG 3'

Dad d<br>Dad r	5' TCGTCGATAAGGAGCGCAAGA 3'<br>5' CGACGCCAGTGCATATGCAA 3'
Ipp d<br>Ipp r	5' GGATTATAGCTGCTGTTTGGAAG 3'<br>5' TACCGCACAGAAACTCCAATAAG 3'
Adar d<br>Adar r	5' CTAGCAAGCCGATGATGAAGTTG 3'<br>5' GGTAAGGTTCACGCGAACCTAA 3'
Hs3st-A Su d<br>Hs3st-A Su r	5'- AGCACGAAAAGTGGCTACAA- 3'<br>5'- CTCGTTATGGGTTAAAGCTGTT- 3
Mpcp d<br>Mpcp r	5’ CTCACCTGATACCTCGTATGC 3’<br>5’ GTTTTGCCGTGGAGAGGTGTA 3’
Prosap d<br>Prosap r	5’ CGTTGGGCTTACAGCTCTAG 3’<br>5’ GCGCAATCCGTCATACAGAAC 3’
cg17090 d<br>cg17090 r	5’ AAACCGGCAGAGTCAGAGTG 3’<br>5’ GAAAAATACCCTTGCGTGTGCT 3’
cg7943 d<br>cg7943 r	5’ CCCGAGGTTTAGGAGGCTAA 3’<br>5’ TGCGTTTAACAGCACTGTCACT 3’
ras d<br>ras r	5'- GAGGGATTCCTGCTCGTCTTCG- 3'<br>5'- GTCGCACTTGTTACCCACCATC- 3'

Supplementary Table 2.

Primer sequences used in RT-qPCR analysis

Primer pair	Sequences
mAcR-60C G d	5'-CTGCTTGGGTATGACCTTGGCGTTG-3'
mAcR-60C G r	5'-TTCAGCCTGCCACTCGGACGG-3'
Rph G d	5'-AACAACTCCGGCAACTCACAAACG-3'
Rph G r	5'-AGGCCTCACGATAGCTAATGGCAA-3'
Syn2 G d	5'-AATGCCAAGTCCTGCAAAGTCTGCTG-3'
Syn2 G r	5'-TATATCATTTTGAGTGTGAGTCCATGCG-3'
Hs3st-A RT d	5'-CAAGGGCTTCTACTGTCTG-3'
Hs3st-A RT r	5'-AAATCGCTGATTGTACTCGG-3'
cg32017 RT d	5'-CGTATTCCATTTGAACACCATC-3'
cg32017 RT r	5'-GCTTCCTTGTATAGCCACTGA-3'
Ras fw	5'-GAGGGATTCCTGCTCGTCTTCG-3'
Ras rev	5'-GTCGCACTTGTTACCCACCATC-3'
